# Quantifying synchrony patterns in the EEG of Alzheimer’s patients with linear and non-linear connectivity markers

**DOI:** 10.1007/s00702-015-1461-x

**Published:** 2015-09-28

**Authors:** Markus Waser, Heinrich Garn, Reinhold Schmidt, Thomas Benke, Peter Dal-Bianco, Gerhard Ransmayr, Helena Schmidt, Stephan Seiler, Günter Sanin, Florian Mayer, Georg Caravias, Dieter Grossegger, Wolfgang Frühwirt, Manfred Deistler

**Affiliations:** AIT Austrian Institute of Technology GmbH, Vienna, Austria; Department of Neurology, Clinical Section of Neurogeriatrics, Graz Medical University, Graz, Austria; Department of Neurology, Innsbruck Medical University, Innsbruck, Austria; Department of Neurology, Vienna Medical University, Vienna, Austria; Department of Neurology and Psychiatry, Linz General Hospital, Linz, Austria; Institute of Molecular Biology and Biochemistry, Graz Medical University, Graz, Austria; Dr. Grossegger and Drbal GmbH, Vienna, Austria; Institute for Mathematical Methods in Economics, Vienna University of Technology, Vienna, Austria

**Keywords:** EEG synchrony markers, Alzheimer’s disease, Compensatory mechanism, Coherence, Granger causality, Canonical correlation

## Abstract

We analyzed the relation of several synchrony markers in the electroencephalogram (EEG) and Alzheimer’s disease (AD) severity as measured by Mini-Mental State Examination (MMSE) scores. The study sample consisted of 79 subjects diagnosed with probable AD. All subjects were participants in the PRODEM-Austria study. Following a homogeneous protocol, the EEG was recorded both in resting state and during a cognitive task. We employed quadratic least squares regression to describe the relation between MMSE and the EEG markers. Factor analysis was used for estimating a potentially lower number of unobserved synchrony factors. These common factors were then related to MMSE scores as well. Most markers displayed an initial increase of EEG synchrony with MMSE scores from 26 to 21 or 20, and a decrease below. This effect was most prominent during the cognitive task and may be owed to cerebral compensatory mechanisms. Factor analysis provided interesting insights in the synchrony structures and the first common factors were related to MMSE scores with coefficients of determination up to 0.433. We conclude that several of the proposed EEG markers are related to AD severity for the overall sample with a wide dispersion for individual subjects. Part of these fluctuations may be owed to fluctuations and day-to-day variability associated with MMSE measurements. Our study provides a systematic analysis of EEG synchrony based on a large and homogeneous sample. The results indicate that the individual markers capture different aspects of EEG synchrony and may reflect cerebral compensatory mechanisms in the early stages of AD.

## Introduction

### Alzheimer’s disease

**Fig. 1:**
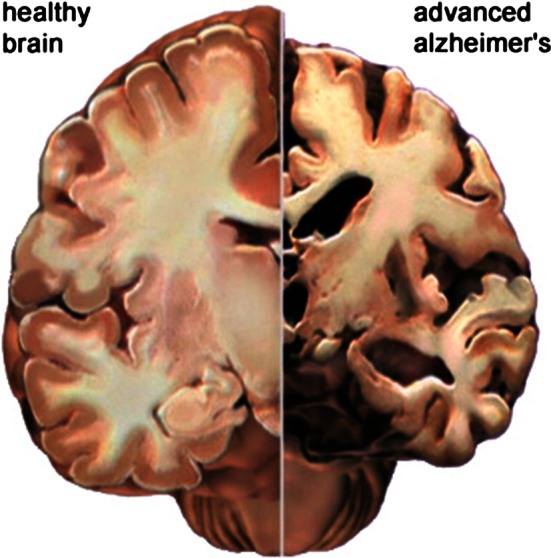
Cerebral slice of a healthy brain and a brain in advanced AD: in AD, shrinkage is especially severe in the hippocampus and ventricles (fluid-filled spaces within the brain) grow larger. Image credit: 2014 Alzheimer’s Association. http://www.alz.org. All rights reserved. Illustrations by Stacy Jannis

Dementia is a disorder of cognitive abilities that has increasing prevalence with age. Alzheimer’s disease (AD) is estimated to account for 60–80 % of dementia cases; hybrid forms with other dementia types occur frequently (Schmidt et al. 2010; Jellinger 2007). AD is a progressive brain disorder that is associated with neuronal cell loss and the development of neurofibrillary tangles and cortical amyloid plaques, e.g., in the hippocampus (Braak et al. 2006). Additionally, alterations in transmitter-specific markers including forebrain cholinergic systems are prevalent in AD (McKhann et al. 2011). Cognitive deficits include impairment of learning and memory, semantic difficulties, deficits in judgement, abstract or logical reasoning, planning and organizing, and, in the late stage of AD, impaired motor functions including chewing and swallowing. As from AD diagnosis, the average survival time ranges from 5 to 8 years (Jeong 2004; Bracco et al. 1994). Figure 1 illustrates the structural cerebral changes that occur in advanced AD.

In Europe, approximately 10.93 million individuals suffered from any form of dementia in 2013. This incidence rate was estimated to increase to 20.75 million by 2050 (Alzheimer’s Disease International 2013). On a global scale, the organization Alzheimer’s Disease International projected the number of dementia cases to increase from 44.35 million in 2013 to 135.46 million by 2050 (Alzheimer’s Disease International 2013). Assuming a prevalence rate of 70 % of AD-caused dementia, the incidence rate of AD would hereby increase from approximately 31 million in 2013 to approximately 95 million by 2050.

Cognitive decline caused by AD entails both severe social and economic consequences (Alzheimer’s Disease International 2010; World Health Organization and Alzheimer’s Disease International 2012). An early diagnosis of the disease is the basis for medical treatment, caregiving, and consultation (Schmidt et al. 2010; Alzheimer’s Disease International 2011). Up to this moment, there is no definite in vivo diagnosis of AD; the disease is classified either as possible or probable AD according to well-defined criteria (McKhann et al. 2011). In clinical practice, obligatory screening for AD includes the assessment of the neurological, internistic, and psychiatric status, neuropsychological tests, a complete blood count, and cerebral magnetic resonance imaging (MRI). Additionally, clinical studies suggest genotyping, liquor analysis, serology, imaging procedures such as positron emission tomography (PET) and functional MRI, as well as the electroencephalogram (EEG) as diagnostic supplements (Schmidt et al. 2010; Laske et al. 2015).

### EEG synchrony in AD patients

One of the major EEG changes that have been reported in AD are perturbations of EEG synchrony (cf. Jeong 2004; Dauwels et al. 2010a for recent reviews). Several studies have analyzed group differences of resting-state EEG synchrony between AD patients, subjects with mild cognitive impairment (MCI), and normal elderly controls: Pearson correlation coefficients were analyzed in Dauwels et al. (2010a), coherences in Locatelli et al. (1998), Wada et al. (1998), Anghinah et al. (2000), Stevens et al. (2001), Adler et al. (2003)^1^, van der Hiele et al. (2007), Jelles et al. (2008), Akrofi et al. (2009), and Dauwels et al. (2010a), partial coherences in Dauwels et al. (2010a), Granger causalities and directed transfer functions in Dauwels et al. (2010a) and Babiloni et al. (2009), information-theoretic measures such as mutual information in Jeong et al. (2001), Wan et al. (2008), and Dauwels et al. (2010a), phase synchrony measures in Stam et al. (2003), Pijnenburg et al. (2004), Stam et al. (2005), Kramer et al. (2007), Stam et al. (2007), Park et al. (2008), Pijnenburg et al. (2008), and Dauwels et al. (2010a), and stochastic event synchrony in Dauwels et al. (2007) and Dauwels et al. (2010a). Most of these studies have suggested a decrease of resting-state EEG synchrony for AD patients as compared to the controls. Additionally, some studies have investigated group differences of EEG synchrony during cognitive tasks (coherences in Hogan et al. 2003; Jiang 2005b; Jiang and Zheng 2006; Hidasi et al. 2007; van der Hiele et al. 2007; Güntekin et al. 2009, and synchronization likelihood in Pijnenburg et al. 2004) or during photic stimulation (coherences in Wada et al. (1998), Kikuchi et al. (2002) and Jiang (2005a)). Especially during working memory tasks, increased EEG synchronies have been reported for MCI subjects (and in a few cases also for AD patients) as compared to the controls (cf. Jiang 2005b; Jiang and Zheng 2006). This phenomenon has been attributed to compensatory mechanisms of the brain (Dauwels et al. 2010b; Smith et al. 2007).

However, there have been only few studies that correlate EEG synchrony measures with AD severity as measured by neuropsychological tests. Studies investigating coherences have reported no significant correlations with the neuropsychological test results, neither in resting state (Adler et al. 2003; Kikuchi et al. 2002) nor during a working memory task (Kikuchi et al. 2002). There have been several studies finding significant correlations between neuropsychological test results and synchronization likelihood (Stam et al. 2003; Pijnenburg et al. 2004; Babiloni et al. 2006), as well as between test results and global field synchronization (Park et al. 2008).

### About this study

The purpose of this study was to derive markers for EEG synchrony from the (multivariate) spectral density and information theory. We investigated whether these markers correlated with AD severity as measured by a neuropsychological test score. Hereby, quadratic synchrony courses were analyzed in order to take compensatory cerebral mechanisms into account. In contrast to most studies, we investigated synchrony not between single EEG channels but between channel groups to gain more robust EEG markers. The study was conducted within a project (No. 827462) funded by a Grant from the Austrian Research Promotion Agency FFG. It has been approved by the ethics committees of the Medical Universities of Graz, Innsbruck and Vienna, and by the ethics committee of Upper Austria.

This paper has been organized in the following way: Sect. 2 is concerned with the materials and methods applied in this study. We describe the sample data, the EEG preprocessing procedure, the markers for EEG synchrony, the estimation of common factors for synchrony, and the methods for analyzing their changes with progressing AD. Section 3 provides the study results. Finally, Sect. 4 discusses the findings and provides concluding remarks.

## Materials and methods

### Study subjects

The study sample consisted of 79 subjects (50 female, 29 male) diagnosed with probable AD according to NINCDS-ADRDA criteria (McKhann et al. 2011). All subjects were participants in the multi-centric cohort study Prospective Dementia Registry Austria (PRODEM-Austria) of the Austrian Alzheimer Society. Enrollment criteria included the availability of a caregiver, written informed consent of each participant and caregiver, as well as the absence of co-morbidities affecting the conduction of the study. Clinical assessments—including EEG recordings—were conducted at the Medical Universities of Graz, Innsbruck, Vienna, and the General Hospital Linz, each of them complying with a homogeneous study protocol. The subjects were aged between 52 and 88 years (mean = 73.57, standard deviation = 9.22) with a duration of probable AD ranging from 2 to 120 months (mean = 25.54, standard deviation = 22.08). Additionally, each subject’s highest completed level of education was classified on a scale of 1 (primary school) to 6 (tertiary institution). Cognitive deficits were evaluated by Mini-Mental State Examination (MMSE) on a scale of 0–30 with lower scores indicating more severe cognitive impairment (Folstein et al. 1975). The study subjects reached MMSE scores between 15 and 26 (mean = 22, standard deviation = 3.16).

### EEG recordings

**Fig. 2:**
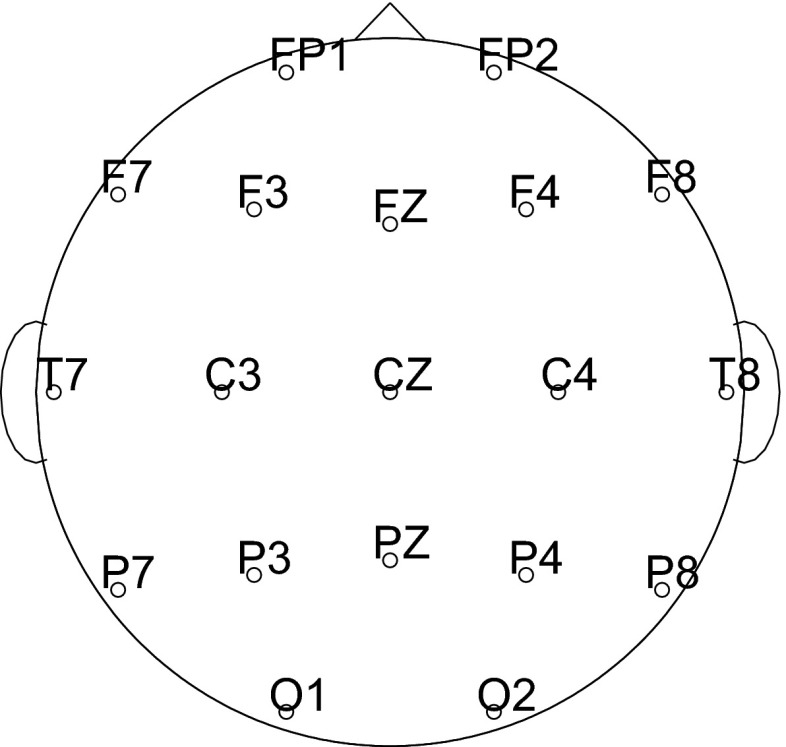
Electrode placement on the scalp as seen from above (Int. 10–20 system)

EEG data were recorded from 19 gold cup electrodes placed according to the International 10–20 system (Jasper 1958). Figure 2 illustrates the electrode placement on the scalp. Connected mastoids were used as reference and the ground electrode was located between channels FZ and CZ. Additionally, both horizontal and vertical electrooculogram (EOG) channels were recorded by electrodes placed above/below the left eye and at the outer corners of both eyes. A wrist clip electrode acquired an electrocardiogram (ECG) channel. The signals were amplified, band-pass (0.3–70 Hz), and notch (50 Hz) filtered by an *AlphaEEG* amplifier (alpha trace medical systems) and digitized at 256 Hz with a resolution of 16 bits. Impedances were kept below 10 k$\Omega$. All four recording sites used identical equipment and software settings for the EEG recordings.

All EEG recording were conducted in accordance with a clinically predefined paradigm consisting of two parts: initially, the subjects were positioned upright in armchairs with integrated neck support in a resting but awake condition with closed eyes (180 s). This was followed by a cognitive task with open eyes where subjects were asked to memorize and recall faces and corresponding names shown on a screen (130 s). This visual-verbal memory test was designed by neurologists especially for dementia patients, as episodic memory and processing of complex stimuli are among the earliest and most frequently impaired cognitive functions in AD. Throughout this work, the recording stages are referred to as resting phase and active phase.

### EEG preprocessing

EEG recordings can be corrupted by electrical signals of non-neuronal origin. These so-called artifacts have either physiological or technical sources. Physiological sources include eye movements and blinking, muscular tension, movement, transpiration, cardiac activity, and talking. Technical artifacts are caused by spurious noise from electronic devices, induction from the mains supply (at 50 or 60 Hz), or poor electrode contacts. EEG preprocessing aims at removing these artifacts and obtaining “pure” neuronal signals. In this study, we applied the following preprocessing steps:

*Pre-selection* At first, EEG segments corrupted by non-removable artifacts, e.g., from poor electrode contacts, were visually identified and excluded from further analyses. On average, 10 % of the resting phase and 35 % of the active phase were excluded, thus leaving an average of 162 s of the resting phase and 84 s of the active phase for our analyses.

*High-pass filtering* The remaining EEG, EOG, and ECG signals were then digitally high-pass filtered using a stable, direct-form finite impulse response (FIR) filter with linear phase, order 340^2^ and a border frequency of 2 Hz. Here any non-neuronal trends and low-frequency artifacts—e.g., from transpiration—were removed from the signals.

*Removing cardiac artifacts* Next, artifacts originating from cardiac activity were approached. These artifacts appear—mostly in multiple EEG channels—as near-periodic spikes, affecting the EEG signals in a broad frequency range due to their non-sinusoidal waveform and the resulting harmonics. The cardiac artifacts were removed by applying the so-called modified Pan-Tompkins algorithm that makes use of the ECG signal for detecting the locations of the cardiac spikes (Waser and Garn 2013).

*Removing ocular artifacts* Eye-induced artifacts from blinking and ocular movements affect the EEG mostly in the frequency range below 10 Hz. These artifacts occur most prominently in the frontal and fronto-temporal EEG channels, and in several cases also in central and even parietal EEG channels. The eye-induced artifacts were removed by utilizing the EOG channels that captured blinking and ocular movements. However, the EOG channels recorded high-frequency neuronal activities as well; hence, the EOG signals were subject to prior low-pass filtering using a stable, direct-form FIR filter with linear phase, order 340 and a border frequency of 12  Hz. Since no dynamic dependences between EOG and EEG were observed, eye-induced artifacts could be removed by applying static linear regression of each EEG signal on the EOG signals.

**Fig. 3:**
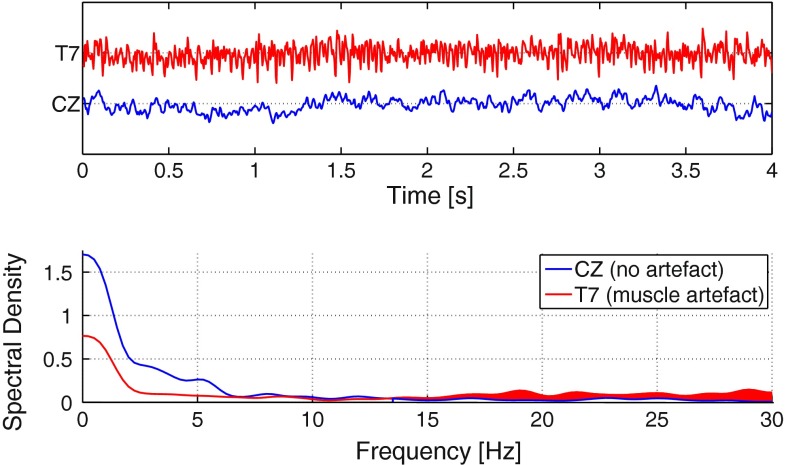
Comparison of an artifact-corrupted channel T7 (*red*) and an artifact-free channel CZ (*blue*) both in time and frequency domain. Muscular induced artifacts altered T7 in a broad frequency range, here illustrated by the *red area*

*Low-pass filtering* Finally, the EEG signals were digitally low-pass filtered using a stable, direct-form FIR filter with linear phase, order 340 and border frequency 15 Hz. In this way, high-frequency artifacts, e.g., from muscle tension, were removed from the EEG. The border frequency of 15 Hz was determined due to the observation that muscular induced artifacts altered the EEG signals from 15 Hz upwards. This is demonstrated in Fig. 3 that shows typical EEG segments of an artifact-corrupted channel T7 (red) and an artifact-free channel CZ (blue) both in time and frequency domain. The artifacts in T7 alter the spectral density in a broad frequency range, here illustrated by the red area. For the benefit of minimizing the presence of artifacts in the preprocessed EEG, the comparably low border frequency of 15 Hz was thus accepted.

*Segmentation* In general, brain dynamics and, consequently, EEG signals are non-stationary (Kaplan et al. 2005). However, since this study’s methods rely on (wide-sense) stationarity of the signals, i.e., a certain invariance of the first and second moments with respect to time, the EEG was divided in “quasi-stationary” 4-s segments with a 2-s overlap. The length of 4 s was the maximum length where EEG segments were still stationary as verified by an augmented Dickey–Fuller test (Dickey and Fuller 1979). All further analyses were carried out on each of these artifact-corrected and band-pass filtered (2–15 Hz) 4-s EEG segments.

### EEG in a stochastic framework

Each EEG segment was interpreted as part of a trajectory of a real-valued stationary stochastic process $\left( x(t)\right)$ with time index $t\in \mathbb {Z}$. Here, $\left( x(t)\right)$ is multivariate, i.e., it consists of subprocesses $\left( x_i(t)\right)$ where the index $i=1,\ldots ,n$ corresponds to the number of EEG channels. We considered stochastic processes with an infinite auto-regressive (AR($\infty$)) representation (see Hannan and Deistler 1988):

1 $$\begin{aligned} \sum _{s=0}^{\infty }{A(s)x(t-s)}=\epsilon (t) \end{aligned},$$

where $A(s)\in \mathbb {R}^{n\times n}$ satisfies $\sum _{s=0}^{\infty }{\Vert A(s)\Vert }<\infty$ and $\epsilon (t)$ is white noise that is orthogonal to $x(t-s)$ for $s>0$. The covariance function of $\left( x(t)\right)$ is defined as $\gamma (s)=\mathbb {E}x(t+s)x(t)'$ with time lag $s\in \mathbb {Z}$. The elements $\gamma _{ij}$ describe the linear dependence between $x_i$ and $x_j$. The (multivariate) spectral density of $\left( x(t)\right)$ at frequencies $\lambda \in \left[ -\pi ,\pi \right]$ is defined as follows:

2 $$\begin{aligned} f(\lambda ) = \frac{1}{2\pi }\sum _{s=-\infty }^{\infty }{e^{-i\lambda s}\gamma (s)} \end{aligned},$$

where the diagonal elements $f_{ii}$ are the auto-spectra of $x_i$ and the off-diagonal elements $f_{ij}$ are the cross-spectra of $x_i$ and $x_j$. We assumed throughout that $f(\lambda )$ has full rank for all $\lambda$. The spectral density $f(\lambda )$, the covariance function $\gamma (s)$ ,and the AR-coefficients *A*(*s*) are closely related to each other. Based on these functions, the following concepts of synchrony for the computation of our EEG markers were employed:

*Coherence* The coherence between $x_i$ and $x_j$ can directly be derived from the cross-spectrum $f_{ij}$ and the respective auto-spectra $f_{ii}$ and $f_{jj}$. It is defined as

3 $$\begin{aligned} C_{ij}(\lambda ) = \frac{|f_{ij}(\lambda )|^2}{f_{ii}(\lambda ) f_{jj}(\lambda )} \end{aligned}$$

with $\lambda \in \left[ -\pi ,\pi \right]$. $C_{ij}$ takes values between 0 and 1 with values close to 1 indicating a strong linear dependence between $x_i$ and $x_j$ (Brillinger 1981). Due to the symmetry $C_{ij}=C_{ji}$, coherence provides no information on the direction of influence. Furthermore, it cannot distinguish direct from indirect dependencies for $n>2$.

*Partial coherence* The idea of partial coherence is to gain information on the direct dependencies between $x_i$ and $x_j$. It is defined as the coherence between the residuals of the orthogonal projections of $x_i$ and $x_j$ on the space that is spanned by the $x_k$ with $k= \left\{ 1,\ldots ,n\right\} \setminus \left\{ i,j\right\}$ (Brillinger 1981). The partial coherence can be derived directly from the inverse of the spectral density $g=f^{-1}$ as

4 $$\begin{aligned} pC_{ij}(\lambda ) = \frac{|g_{ij}(\lambda )|^2}{g_{ii}(\lambda ) g_{jj}(\lambda )} \end{aligned}$$

with $\lambda \in \left[ -\pi ,\pi \right]$ (Dahlhaus 2000). $pC_{ij}$ takes values between 0 and 1 with higher values indicating stronger direct linear dependence between $x_i$ and $x_j$. The partial coherence is again symmetric and, thus, provides no information on the direction of influence either.

*Phase shift* The cross-spectral density $f_{ij}$ between $x_i$ and $x_j$ is a complex valued function. In polar coordinates, it can be rewritten as

5 $$\begin{aligned} f_{ij}(\lambda ) = |f_{ij}(\lambda )|e^{i\Phi _{ij}(\lambda )}. \end{aligned}$$

Here, $\Phi _{ij}(\lambda )$ measures the expected phase shift between $x_i$ and $x_j$ at frequency $\lambda$. The normalized phase $n\Phi _{ij} = |\Phi _{ij}|/\pi$ can be used as a symmetric measure for synchrony that takes values between 0 and 1.

*Granger causality* Granger introduced the concept of Granger causality for investigating not only the dependencies between $x_i$ and $x_j$, but also the direction of these dependencies (Granger 1969). The idea is that if knowledge of $x_i(t-s)$ with $s>0$ improves the prediction of $x_j(t)$, then $x_i$ is said to be Granger causal for $x_j$. Considering the bivariate AR($\infty$) representation

6 $$\begin{aligned} \sum _{s=0}^{\infty }{A(s)\left( \begin{array}{c}x_i(t-s)\\ x_j(t-s)\end{array}\right) }=\epsilon (t) \end{aligned}$$

with $A(s)\in \mathbb {R}^{2\times 2}$, Granger non-causality of $x_i$ for $x_j$ is equivalent to (cf. Eichler 2006)

7 $$\begin{aligned} A_{ji}(s)=0\ \quad \forall s=1,\ldots ,\infty . \end{aligned}$$

Although Granger causality provides information on the direction of dependence, it does not take any other subprocesses but $x_i$ and $x_j$ into account.

*Conditional Granger causality* The concept of conditional Granger causality is a generalization of the original bivariate version. The idea is that if knowledge of the past $x_i$ improves the prediction of $x_j$ given $x_k$ with $k=\left\{ 1,\ldots ,n\right\} \setminus \left\{ i,j\right\}$, then $x_i$ is said to be conditionally Granger causal for $x_j$ (Flamm et al. 2012). Conditional Granger non-causality is equivalent to (cf. Eichler 2006)

8 $$\begin{aligned} A_{ji}(s)=0\ \quad \forall s=1,\ldots ,\infty , \end{aligned}$$

where the $A(s)\in \mathbb {R}^{n\times n}$ are the coefficients of the AR($\infty$)-representation (1).

*Canonical correlation* All presented concepts aim at quantifying the dependence between two univariate processes $x_i(t)$ and $x_j(t)$. Hotelling introduced the concept of canonical correlation analysis in order to analyze the dependence between multivariate $x_I(t)\in \mathbb {R}^p$ and $x_J(t)\in \mathbb {R}^q$ with disjoint index sets $I,J\subset \left\{ 1,\ldots ,n\right\}$ (Hotelling 1936). The idea is to determine, in a first step, those linear transformations $a_1\in \mathbb {R}^p$ and $b_1\in \mathbb {R}^q$ that maximize $\rho _1=corr\left( a_1'x_I(t),b_1'x_J(t)\right)$. Next, the $a_2\in \mathbb {R}^p$ and $b_2\in \mathbb {R}^q$ maximizing $\rho _2=corr\left( a_2'x_I(t),b_2'x_J(t)\right)$ with side conditions $a_2'x_I(t)\bot a_1'x_I(t)$ and $b_2'x_J(t)\bot b_1'x_J(t)$ are determined. Repeating this procedure $r=\min {\left( p,q\right) }$ times defines the canonical correlation coefficients $\rho _1\ldots \rho _r$. They are the eigenvalues of

9 $$\begin{aligned} \gamma _{II}^{-\frac{1}{2}} \gamma _{IJ} \gamma _{JJ}^{-1} \gamma _{JI} \gamma _{II}^{-\frac{1}{2}}, \end{aligned}$$

where $\gamma _{II}$ and $\gamma _{JJ}$ are the auto-covariances and $\gamma _{IJ}=\gamma _{JI}'$ are the cross-covariances of $x_I(t)$ and $x_J(t)$. The canonical correlation coefficients provide information on the symmetric, i.e., non-directional, linear dependence between $x_I(t)$ and $x_J(t)$. They are, however, designed to capture time-static dependencies only.

*Dynamic canonical correlation* Brillinger introduced dynamic canonical correlation analysis as a generalization of the original time-static version in Brillinger (1981). The dynamic canonical correlation coefficients $\rho _1(\lambda )\ldots \rho _r(\lambda )$ are defined as maximum correlation between $\sum _s{a_i(t-s)'x_I(t)}$ and $\sum _s{b_i(t-s)'x_J(t)}$ with $a_i\in \mathbb {R}^p$ and $b_i\in \mathbb {R}^q$. They are the eigenvalues of

10 $$\begin{aligned} f_{II}^{-\frac{1}{2}}(\lambda ) f_{IJ}(\lambda ) f_{JJ}^{-1}(\lambda ) f_{JI}(\lambda ) f_{II}^{-\frac{1}{2}}(\lambda ) \end{aligned}$$

at frequencies $\lambda$, where $f_{II}$ and $f_{JJ}$ are the auto-spectra and $f_{IJ}=\overline{f_{JI}'}$ are the cross-spectra of $x_I(t)$ and $x_J(t)$. Thus, the dynamic canonical correlation coefficients provide information on the symmetric dynamic linear dependence between $x_I$ and $x_J$.

*Cross-mutual information* The previous concepts aim at measuring linear dependences only. However, the complexity of neuronal processes may suggest the implementation of markers for non-linear synchrony. Shannon and Weaver introduced the concept of cross-mutual information for measuring the information content transmitted between two systems in Shannon and Weaver (1949). The cross-mutual information between two discrete random variables *X* and *Y* measures the amount of information that can be obtained about one random variable by observing the other random variable. It is defined as

11 $$\begin{aligned} cMI(X,Y) = \sum _{x,y}{p_{XY}(x,y) \log _2{\frac{p_{XY}(x,y)}{p_{X}(x)p_{Y}(y)}}}, \end{aligned}$$

where *x* and *y* are the observations of *X* and *Y* with joint probability distribution $p_{XY}$ and marginal probability distributions $p_{X}$ and $p_{Y}$, respectively. The cross-mutual information is symmetric and provides information on non-linear couplings.

### Computation of EEG synchrony markers

**Fig. 4:**
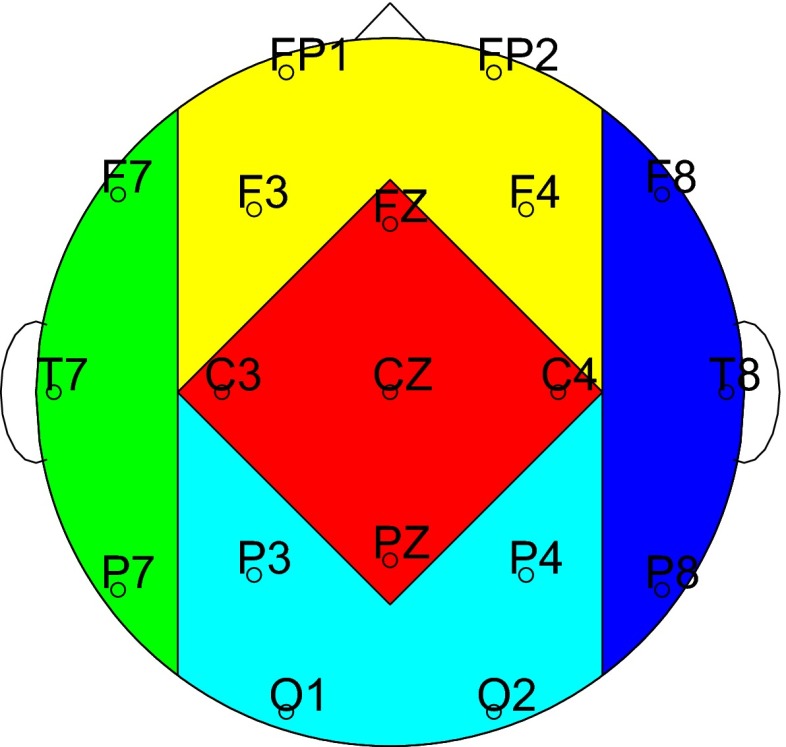
Distribution of EEG clusters (cf. Dauwels et al. 2010b): Anterior (*yellow*), temporal/left (*green*), Central (*red*), temporal/right (*blue*), and Posterior (*cyan*)

The presented concepts were employed for the computation of EEG synchrony markers. Since single EEG channels are—despite EEG preprocessing—prone to disturbances, we arranged the EEG channels into clusters and analyzed the synchrony between these clusters in order to obtain more robust results. The following five clusters were defined (cf. Dauwels et al. 2010b; Waser et al. 2014): Anterior (FP1, FP2, F3, F4), Temporal/Left (F7, T7, P7), Central (FZ, C3, CZ, C4, PZ), Temporal/Right (F8, T8, P8), and Posterior (P3, P4, O1, O2). Figure 4 shows the distribution of these clusters on the scalp.

**Fig. 5:**
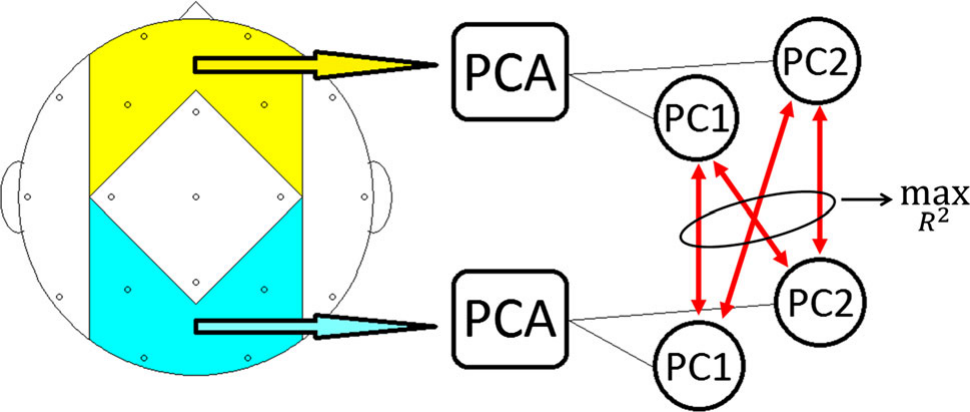
Diagram of synchrony analysis between Anterior (*yellow*) and Posterior (*cyan*) clusters: Step 1: PCA is performed for each cluster; Step 2: Each synchrony marker (indicated by *red arrows*) is calculated between the first two PCs of the Anterior cluster and the first two PCs of the Posterior cluster; Step 3: Maximize correlation between synchrony marker and MMSE in terms of coefficient of determination $R^2$

In our 19-channel recording framework, this clustering reflects roughly the position of the big cerebral lobes. For each cluster, we conducted principal component analysis (PCA) and investigated the synchrony between the principal components (PC) of two clusters under consideration. To be more precise, we investigated synchrony between the first two PCs of one cluster and the first two PCs of another cluster, since the first two PCs together accounted for over 90 % of the variability in the respective channel data. We then used the PC combination where the synchrony marker related the most (in terms of coefficient of determination $R^2$) with MMSE scores. This was done for all ten cluster combinations (cf. Fig. 4). Figure 5 illustrates the described approach by taking the example of synchrony between the Anterior (yellow) and Posterior (cyan) clusters. The red arrows indicate the measurement of synchrony. The idea of using the first two PCs was to use only the “main” information common to all channels of a cluster in order to be robust against irregularities in single EEG channels. This method has already been demonstrated in Garn et al. (2015, 2014); Waser et al. 2013). Static and dynamic canonical correlations were calculated directly between the clusters without previous PCA (Waser et al. 2014). A more common approach than our PCA method is the computation of marker averages between each channel of one cluster and each channel of another cluster (c.f. Dauwels et al. 2010b), which is best suited for high-density EEG recordings. For EEG recordings with a low number of channels and, hence, only few marker values, averaging could be misleading if there were irregularities in one or more EEG channels. However, we also computed averages in order to compare the performance of our PCA approach with this more commonly used technique.

We estimated the spectral density by using an indirect estimation procedure. The sample covariance $\widehat{\gamma }$ was tapered—i.e., component-wise multiplied—with a lag-window *w*(*s*) that attenuated $\widehat{\gamma }$ for higher lags. The tapering was employed to reduce the leakage effect and to construct a consistent spectral estimate (Tukey 1967). We used a Parzen window in order to ensure a positive semi-definite estimate of the spectral density (Parzen 1962). The tapered sample covariances were then Fourier transformed, resulting in the estimate

12 $$\begin{aligned} \widehat{f}(\lambda )=\frac{1}{2\pi }\sum _{s=-c}^{c}{w(s)\widehat{\gamma }(s)\ e^{-i\lambda s}}, \end{aligned}$$

where *c* was the truncation point that determined the number of covariance lags included in $\widehat{f}$. We identified an optimal truncation point of $c=255$ visually by window closing (Tukey 1967), aiming at a smooth spectral estimator that still displayed characteristic spectral peaks such as the individual alpha frequency during resting phases.

The estimates $\widehat{\gamma }$ and $\widehat{f}$ were then used to compute the EEG synchrony markers. Coherences and partial coherences were derived directly from $\widehat{f}$ by using the definitions (3) and (4). In order to study ordinary and conditional Granger causality, estimates $\widehat{A}(s)$ for the AR-coefficients were obtained by solving the Yule–Walker equations (Yule 1927; Walker 1931). The model order was decided by the Akaike information criterion (Akaike 1974). The “degree” of (conditional) Granger causality between cluster *i* and *j* was then determined by using the Euclidean norm of all $\widehat{A}_{ji}(s)$ for fixed *i* and *j*, $s=1,\ldots$. The respective static and dynamic canonical correlation coefficients were derived from $\widehat{\gamma }$ and $\widehat{f}$ by using (9) and (10), respectively. We employed the Euclidean norm of these canonical correlation coefficients as synchrony marker. For the cross-mutual information, joint and marginal probability distribution functions were estimated from the joint data histogram with a $10\times 10$ grid of bins. We used the normalized version of the cross-mutual information that was introduced in Maes et al. (1997).

Markers for Granger causality, conditional Granger causality, canonical correlation, and cross-mutual information were computed in time domain, whereas markers for coherence, partial coherence, phase shift, and dynamic canonical correlation were computed in frequency domain at each frequency and averaged over the frequencies within four bands: $\delta$ from 2 to 4 Hz, $\theta$ from 4 to 8 Hz, $\alpha$ from 8 to 13 Hz, and $\beta _0$ from 13 to 15 Hz. Since different EEG frequencies correspond to different cognitive states, dividing them in bands is a well-established procedure in EEG analysis (cf. Jeong 2004). Table 1 summarizes the EEG synchrony markers (with a shorter notation) that were applied in this study, and the type of synchrony they describe.

**Table 1:** EEG markers and their synchrony characteristics

EEG markers	Synchrony characteristics
Coherence *C*	Linear, symmetric, direct and indirect, bivariate
Partial coherence pC	Linear, symmetric, direct, bivariate
Phase shift $n\Phi$	Linear, symmetric, direct and indirect, bivariate
Granger causality *G*	Linear, asymmetric, direct and indirect, bivariate
Conditional granger causality cG	Linear, asymmetric, direct, bivariate
Canonical correlation $\rho ^c$	Linear, symmetric, direct and indirect, multivariate
Dynamic canonical correlation $d\rho ^c$	Linear, symmetric, direct and indirect, multivariate
Cross-mutual information cMI	Non-linear, symmetric, direct and indirect, bivariate

### EEG synchrony versus AD severity

We analyzed the change of the EEG synchrony markers in the course of AD with quadratic ordinary least squares regression models. In these models, the MMSE score—as a measure for AD severity—was employed as independent variable and each EEG marker as respective dependent variable. Quadratic model functions were used since non-monotonic changes of EEG synchrony due to compensatory neuronal mechanisms have been reported (cf. Park et al. 2008; Smith et al. 2007). The subjects’ age, sex, AD duration, and highest level of completed education were introduced as co-variables. Hereby, age and AD duration were introduced via both linear and quadratic terms. The significance of the regression models was evaluated by Fisher’s *F*-test ($p<0.05$). The goodness of fit was quantified by the coefficient of determination $R^2$. Since several hypotheses were tested on the same sample data, we employed Bonferroni post-correction to control the familywise error rate (cf. Hsu 1996). Thereby, since we computed 8 different EEG markers, the significance level was adjusted from $p=0.05$ to $p=\frac{0.05}{8}=0.00625$. This rather strict correction method was used in order to rule out any spurious testing effects.

### Common factors for EEG synchrony

Since several of the presented EEG synchrony markers are closely related to each other, they could be reflecting the behavior of a small number of unobserved synchrony factors. We used a maximum likelihood approach for estimating these common factors as proposed by Lawley (1940). In order to interpret the factor model, we rotated the factors based on the oblimin criterion, which is a standard method for optimizing the rotation that allows the factors to be oblique (i.e., correlated) (Carroll 1953). In the same way as described in Sect. 2.6, quadratic regression with factor scores as dependent variable, MMSE as independent variable and age, sex, AD duration, and highest level of completed education as co-variables was applied for estimating the relation between the synchrony factors and AD severity.

## Results

In recapitulation, the following 8 EEG markers were analyzed in a resting and a cognitively active phase: coherence *C*, partial coherence *pC*, phase shift $n\Phi$, Granger causality *G*, conditional Granger causality *cG*, canonical correlation $\rho ^c$, dynamic canonical correlation $d\rho ^c$ ,and cross-mutual information *cMI*. Each marker was computed between each of the EEG clusters (cf. Fig. 4): Anterior-Central (A-C), Anterior-Posterior (A-P), Anterior-Temporal/Left (A-TL), Anterior-Temporal/Right (A-TR), Central-Posterior (C-P), Central-Temporal/Left (C-TL), Central-Temporal/Right (C-TR), Posterior-Temporal/Left (P-TL), Posterior-Temporal/Right (P-TR), and Temporal/Left-Temporal/Right (TL-TR). The direction of the asymmetric Granger causality measures *G* and *cG* will be indicated by arrows “$\rightarrow$” and “$\leftarrow$”. *C*, *pC*, $n\Phi$ ,and $d\rho ^c$ were studied frequency bandwise in $\delta$, $\theta$, $\alpha$ and $\beta _0$. We employed quadratic regression models to analyze changes of the EEG synchrony markers with progressing AD as quantified by MMSE scores. As a result, p-values of Fisher’s *F*-tests—the significance levels were Bonferroni corrected from 0.05 to $\frac{0.05}{8}=0.00625$—and coefficients of determination $R^2$ were determined. Additionally, common factor analysis was applied in order to determine the behavior of unobserved underlying synchrony changes.

### Resting phase

**Fig. 6:**
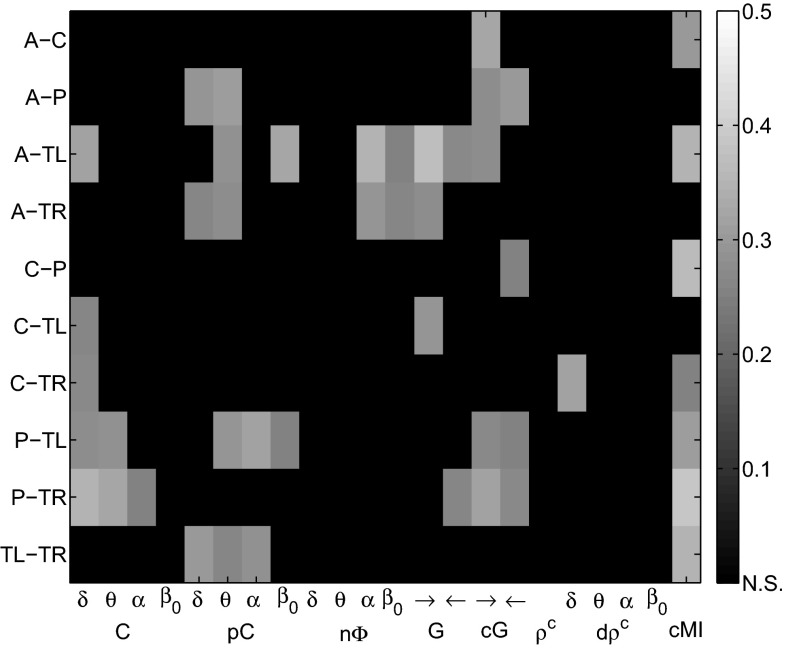
EEG synchrony versus AD severity in the resting phase: coefficients of determination $R^2$ for the quadratic regression models on all cluster pairs. Significant values are *gray-value* coded and non-significant values are indicated by *black fields*

*EEG markers versus MMSE scores* In the resting phase, coherences were significantly related with MMSE scores for several cluster pairs, most prominently between A-TL ($R^2=0.314$ in $\delta$), P-TL ($R^2=0.277$ in $\delta$ and $R^2=0.286$ in $\theta$), and P-TR ($R^2=0.344$ in $\delta$, $R^2=0.321$ in $\theta$ and $R^2=0.257$ in $\alpha$). The partial coherences markers reached $R^2$ values greater than 0.3 between A-P ($R^2=0.311$ in $\theta$), A-TL ($R^2=0.326$ in $\beta _0$), P-TL ($R^2=0.318$ in $\alpha$), and TL-TR ($R^2=0.302$ in $\delta$). The phase shift related significantly with MMSE scores only between A-TL with $R^2=0.350$ in $\alpha$ and A-TR. The Granger causality marker was most significant between A-TL, reaching $R^2=0.374$ for A$\rightarrow$TL. Conditional Granger causalities showed significant relations with MMSE scores in both direction of A-P, P-TL, and P-TR, and a maximum of $R^2=0.327$ between A-C. No significant changes of the canonical correlation marker with increasing AD severity were observed. The dynamic canonical correlation marker related only between C-TR ($R^2=0.316$ in $\delta$) significantly with the MMSE. The cross-mutual information reached $R^2$ values greater than 0.3 between A-C ($R^2=0.303$), A-TL ($R^2=0.351$), C-P ($R^2=0.360$), P-TL ($R^2=0.306$), P-TR ($R^2=0.386$), and TL-TR ($R^2=0.345$). Figure 6 shows the $R^2$ values of the synchrony markers in the resting phase for all cluster combinations as gray color image. Each image pixel corresponds to a synchrony measure (abscissa) at a certain cluster pair (ordinate). Significant $R^2$ values are gray-coded and non-significant values are indicated by black fields. The exact $R^2$ values are provided in Table 2.

**Fig. 7:**
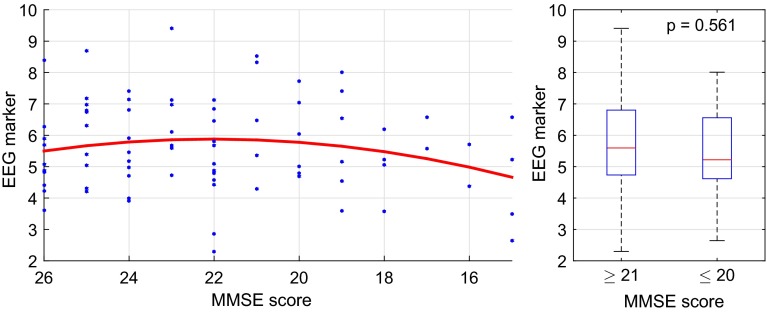
Conditional Granger causality between A$\rightarrow$C in the resting phase: each *blue dot* represents the marker value for one patient and the *red line* illustrates the quadratic regression function with $R^2=0.327$ and $p<0.001$

We observed several significant relations of EEG markers with MMSE scores. For diagnostic purposes, steep monotonic relations with high $R^2$ values would be desirable. However, the relations we found were, in most cases, of non-monotonic nature. The left plot in Fig. 7 shows a scatter plot of the conditional Granger causality between A$\rightarrow$C. Each blue dot represents the marker value for one patient on the ordinate at the corresponding MMSE score on the abscissa. Since the abscissa is reversed, points further to the right correspond to more severe cognitive deficits. The red line illustrates the quadratic regression function that was fitted to the data ($R^2=0.327$, $p<0.001$). Here the regression model function describes an initial increase of the synchrony marker for MMSE scores from 26 to 22, and a decrease from 22 downwards. For the right plot of Fig. 7, we distinguished two regimes of MMSE, i.e., above ($n=55$) and below MMSE scores of 21 ($n=24$). The null hypothesis that the two groups came from the same population (i.e., they have equal medians) was then tested by a Mann–Whitney *U* test (Mann and Whitney 1947). This hypothesis was accepted with $p=0.561$.

**Fig. 8:**
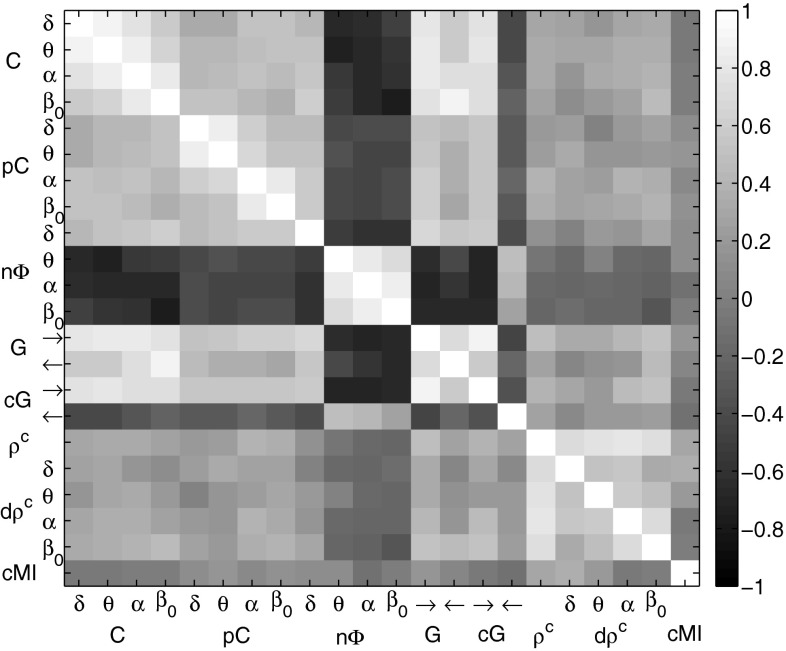
Relation between EEG synchrony markers between A-TL in the resting phase: correlation coefficients as *gray color* image

*Common factors for EEG synchrony* Several of the presented EEG synchrony markers are based on similar concepts. We analyzed whether this conceptual similarity was reflected by the results or, in other words, how the EEG markers correlated with each other. Figure 8 shows the Pearson correlation coefficients of the synchrony markers with each other between A-TL in the resting phase as gray color image. Each image pixel corresponds to a pair of synchrony measures. Bright gray pixels correspond to a positive correlation close to 1 and dark gray pixels correspond to a negative correlation close to $-1$. In this example, interesting correlation patterns were observed. Non-surprisingly, coherences and partial coherences were positively correlated to each other. In addition, positive relation of these measures with Granger causalities was observed. The marker for phase shift, however, was negatively correlated with the coherence measures. Canonical correlations, dynamic canonical correlations, and the cross-mutual information marker were only weakly correlated with each other and the other measures.

**Fig. 9:**
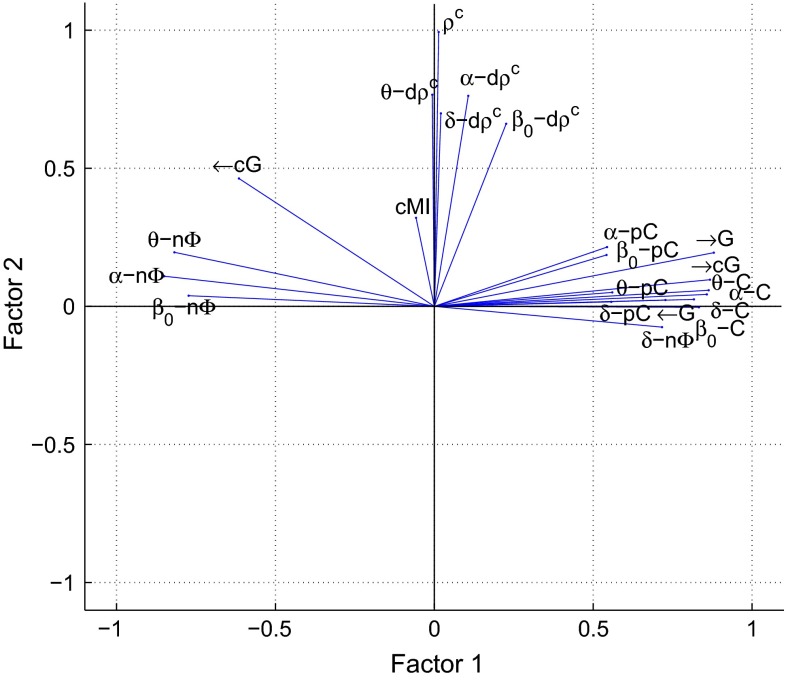
Biplot of the first two factors for synchrony between A-TL in the resting phase: the factors were estimated via ML estimation and rotated with an oblique promax rotation. Each *blue line* corresponds to the loadings of an EEG marker

These correlation patterns could be reflecting the behavior of a low number of unobserved synchrony factors. We estimated these common factors by a maximum likelihood approach and rotated them by using an oblique promax rotation based on the oblimin criterion. Figure 9 shows a biplot of the first two common factors for synchrony between A-TL in the resting phase. The abscissa corresponds to the first factor and the ordinate to the second factor. Although the coordinate axes are shown as orthogonal lines, the factors are correlated with each other (Pearson correlation coefficient of 0.312). The lines represent the observed synchrony markers and their magnitude and sign illustrate how each marker is represented in terms of the common factors. Here the coherences *C*, partial coherences *pC*, Granger causalities *G*, conditional Granger causalities *cG* (in direction $\rightarrow$), and the phase shift $n\Phi$ in $\delta$ contribute positively to the first factor, whereas the phase shift in the remaining frequency bands contributes in a negative way. The second factor represents the canonical correlation $\rho ^c$, the dynamic canonical correlation $d\rho ^c$ in all frequency bands and, although more weakly, the cross-mutual information *cMI*. Interestingly, the conditional Granger causality *cG* (in direction $\leftarrow$) cannot be conclusively assigned to one of the factors.

**Fig. 10:**
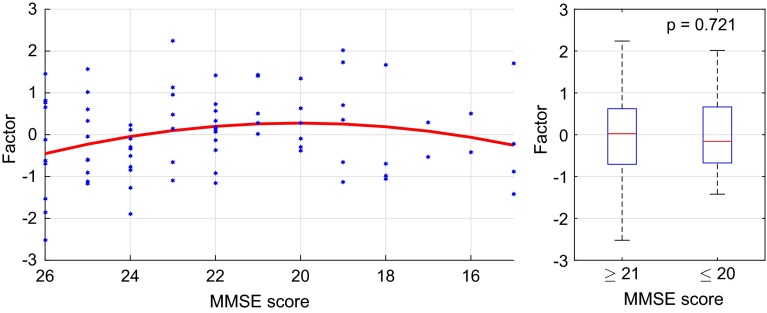
The first common factor between A-TL in the resting phase: the *blue dots* represent the marker values for each patient and the *red line* illustrates the quadratic regression function with $R^2=0.353$ and $p<0.001$

In the context of this study, the next logical step was to investigate how these common factors were related to AD severity. Therefore, we applied the same quadratic regression procedure as for the synchrony markers to each common factor. In the resting phase, the first factors displayed the strongest relation (measured in $R^2$) with MMSE scores, most significantly between A-TL ($R^2=0.353$), C-TL ($R^2=0.332$), and P-TL ($R^2=0.271$). Figure 10 shows a scatter plot of the first common factor between A-TL. As before, each blue dot represents the factor value for one patient on the ordinate at the corresponding MMSE score on the abscissa. Points further to the right correspond to more severe cognitive deficits. The red line illustrates the quadratic regression function that was fitted to the data ($R^2=0.353$, $p<0.001$). As for the individual synchrony markers, the first common factor scores follow an ambiguous trend with an increase for MMSE scores from 26 to 20 and a decrease below that. Again, the slopes of the regression function are rather flat. As before, we distinguished the two regimes of MMSE above ($n=55$) and below 21 ($n=24$) and tested the null hypothesis that the two groups came from the same population by using the Mann–Whitney *U* test. Again, no significant difference was found between the two groups ($p=0.721$).

### Active phase

**Fig. 11:**
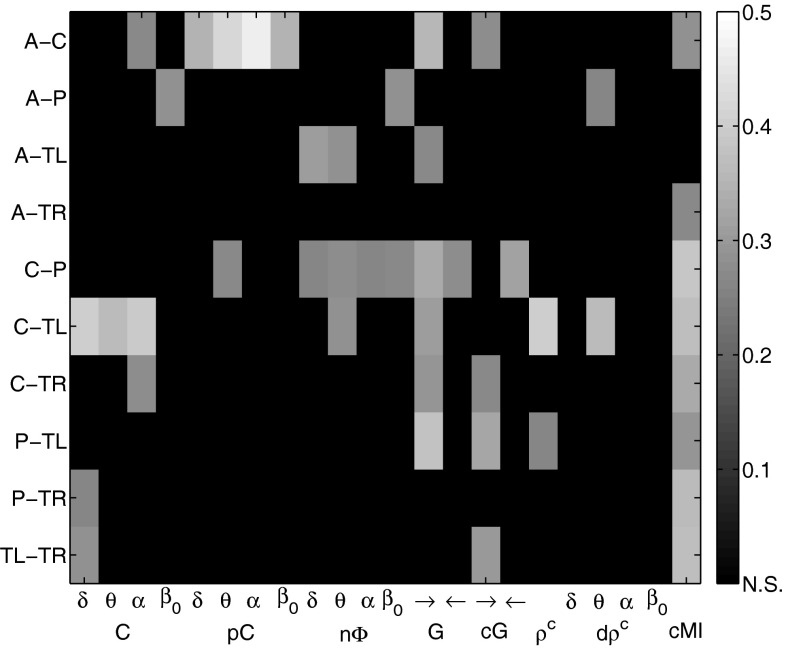
EEG synchrony versus AD severity in the active phase: coefficients of determination $R^2$ for the quadratic regression models on all cluster pairs. Significant values are *gray-value* coded, and non-significant values are indicated by *black fields*

*EEG markers versus MMSE scores* In the active phase, coherences were strongly related to MMSE scores between C-TL with $R^2=0.399$ in $\delta$, $R^2=0.366$ in $\theta$ , and $R^2=0.393$ in $\alpha$. Partial coherences showed the most significant results of all synchrony markers between A-C with $R^2=0.344$ in $\delta$, $R^2=0.420$ in $\theta$, $R^2=0.462$ in $\alpha$ , and $R^2=0.350$ in $\beta _0$. The phase shift, on the other hand, related most significantly with AD severity between A-TL ($R^2=0.310$ in $\delta$) and, especially, C-P in all frequency bands. Both the Granger and the conditional Granger causality markers reached $R^2$ values greater than 0.3: Granger causalities between A-C ($R^2=0.354$), C-P ($R^2=0.329$), C-TL ($R^2=0.307$), and, most prominently, between P-TL with $R^2=0.377$; conditional Granger causalities between C-P ($R^2=0.316$), P-TL ($R^2=0.321$), and TL-TR ($R^2=0.304$). Highly significant results between C-TL were observed for the canonical correlation marker with $R^2=0.402$, and for the dynamic canonical correlations in $\theta$ with $R^2=0.366$. Cross-mutual information related strongly with MMSE scores for eight out of ten cluster pairs, most prominently between C-P ($R^2=0.386$) and C-TL ($R^2=0.373$). Figure 11 shows the $R^2$ values of the synchrony markers in the active phase for all cluster combinations as gray color image. As before, significant values are gray-value coded and non-significant values are indicated by black fields. The exact $R^2$ values are provided in Table 3.

**Fig. 12:**
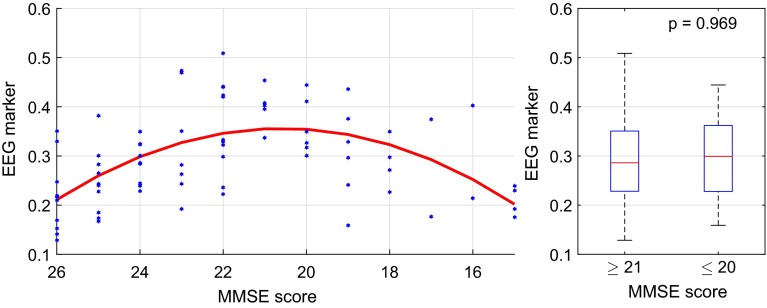
Coherence in $\delta$ between C-TL in the active phase: the *blue dots* represent the marker values for each patient and the *red line* illustrates the quadratic regression function with $R^2=0.399$ and $p<0.001$

In the active phase, $R^2$ values up to 0.462 were observed. However, the regression function fitted to the data were even more ambiguous than in the resting phase. Figure 12 shows a scatter plot of the coherence in $\delta$ between C-TL. Each blue dot represents the marker value for one patient on the ordinate at the corresponding MMSE score on the abscissa. Again, points further to the right correspond to more severe cognitive deficits. The red line illustrates the quadratic regression function that was fitted to the data ($R^2=0.399$, $p<0.001$). The model function describes an initial increase of the synchrony marker for MMSE scores from 26 to 21, and a decrease from 20 downwards. The slopes are steeper than in the resting phase. For the right plot of Fig. 12, the null hypothesis of equal medians of the two groups could clearly not be rejected with $p=0.969$.

**Fig. 13:**
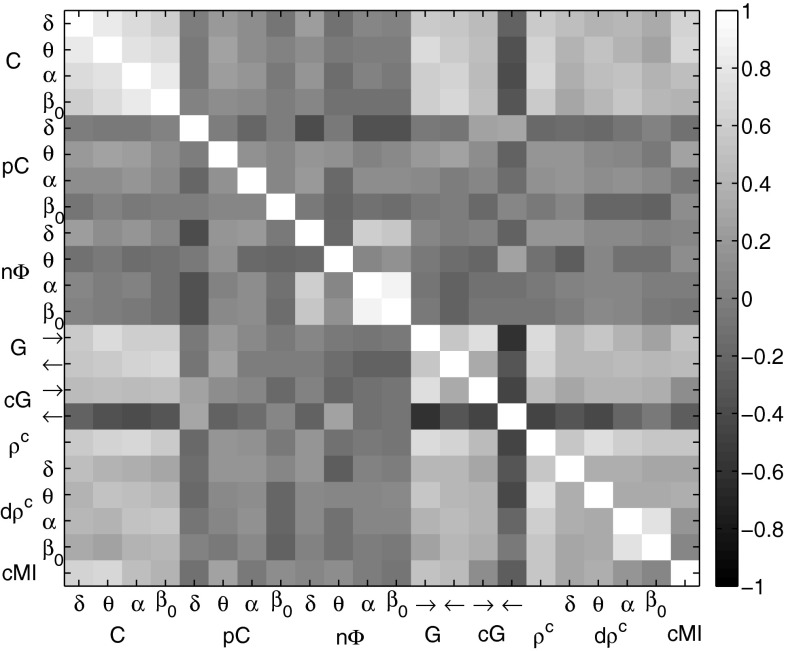
Relation between EEG synchrony markers between C-TL in cognitive phase: correlation coefficients as *gray color* image

*Common factors for EEG synchrony* Next, we analyzed how these EEG markers were related to each other with the objective of identifying synchrony patterns. Figure 13 shows the Pearson correlation coefficients of the markers with each other between C-TL in the active phase as gray color image. Each image pixel corresponds to a pair of synchrony measures. Bright gray pixels correspond to a positive correlation close to 1 and dark gray pixels correspond to a negative correlation close to $-1$. Here coherences, Granger causalities, conditional Granger causalities (in direction $\rightarrow$), canonical correlations, dynamic canonical correlations, and cross-mutual information formed a group of positively correlated markers. Partial coherences and the phase shift showed little correlation with the other measures, whereas conditional Granger causalities in direction $\leftarrow$ were negatively related with the members of the former group.

**Fig. 14:**
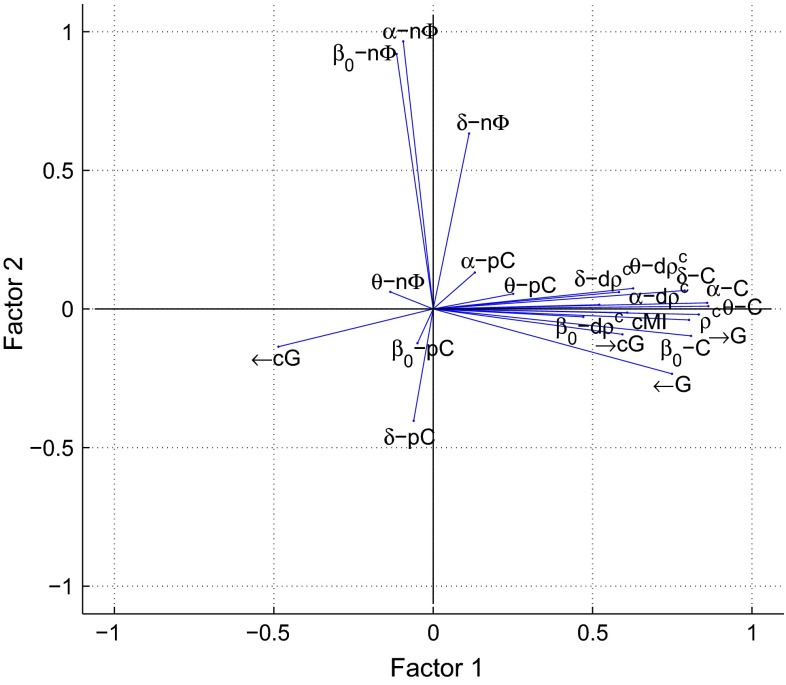
Biplot of the first two factors for synchrony between C-TL in the active phase: the factors were estimated via ML estimation and rotated with an oblique promax rotation. Each *blue line* corresponds to the loadings of an EEG marker

These synchrony patterns in the active phase are represented in Fig. 14 as well, where a biplot of the first two common factors between C-TL is shown. The abscissa corresponds to the first factor and the ordinate to the second factor. The factors are weakly correlated with each other (Pearson correlation coefficient of 0.077). The lines represent the observed synchrony markers and their magnitude and sign illustrate how each marker is represented in terms of the common factors. Here the coherences *C*, Granger causalities *G*, conditional Granger causalities *cG* (in direction $\rightarrow$), canonical correlations $\rho ^c$, dynamic canonical correlations $d\rho ^c$ , and the cross-mutual information *cMI* contribute positively to the first factor, whereas the conditional Granger causality *cG* (in direction $\leftarrow$) contributes in a negative way. The second factor is determined most positively by the phase shift $n\Phi$, and most negatively by the partial coherences *pC* in $\delta$ and $\beta _0$.

**Fig. 15:**
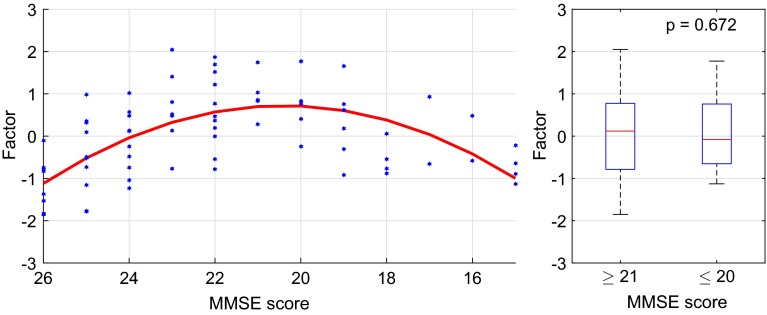
The first common factor between C-TL in the active phase: the *blue dots* represent the marker values for each patient and the *red line* illustrates the quadratic regression function with $R^2=0.433$ and $p<0.001$

The first common factor showed the most significant relations with MMSE scores in the active phase as well. The highest coefficients of determination were observed between C-TL ($R^2=0.433$) and C-TR ($R^2=0.302$). On the left side, Fig. 15 shows a scatter plot of the first common factor between C-TL. As before, each blue dot represents the factor value for one patient on the ordinate at the corresponding MMSE score on the abscissa. Points further to the right correspond to more severe cognitive deficits. The red line illustrates the quadratic regression function that was fitted to the data ($R^2=0.433$, $p<0.001$). Here the first common factor scores followed an ambiguous trend with an increase for MMSE scores from 26 to 21 and a decrease from 20 downwards. Here the reversed U-shape of the regression curve was much more distinct than in the resting phase. The two groups in the right plot of Fig. 15 above ($n=55$) and below MMSE scores of 21 ($n=24$) could not be statistically distinguished by Mann-Whitney U test; the hypothesis of equal medians was accepted with $p=0.672$.

### Comparison: PCA approach versus averaging

We computed synchrony between the electrode clusters by computing it between their first and second PCs. A more common approach is the computation of marker averages between each channel of one cluster and each channel of another cluster. We therefore computed averages as well in order to compare the performance of our PCA approach with this more commonly used technique. In the resting phase, a similar performance of the two methods was observed. The averaging technique reached several $R^2$ values greater than 0.3 and up to 0.372 (cross-mutual information between A-TL). Although significant results were found for mostly the same cluster combinations, $R^2$ values of our PCA approach were on average greater by 0.06. In the active phase, the same cluster combinations were most significant: C-TL (coherences, canonical correlation), A-C (partial coherences), C-P (phase shift), and several combinations for the cross-mutual information. $R^2$ values greater than 0.4 and up to 0.451 were observed. Again, the PCA approach performed on average better in its $R^2$ values by 0.08. In both resting and active phases, the regression models for both methods displayed the same ambiguous trends.

## Discussion and conclusion

### Summary

We analyzed and compared different EEG synchrony markers in AD patients both in a resting and a cognitively active state and how these markers changed with AD severity as measured by MMSE scores. During the resting phase, synchrony between Anterior-Temporal/Left, Posterior-Temporal/Left and Posterior-Temporal/Right EEG channel groups was most significantly related with AD severity. Here coherences, partial coherences, phase shift, Granger causalities, conditional Granger causalities, and the cross-mutual information reached $R^2$ values greater than 0.3 and up to 0.386. None or only weak relations with MMSE scores were observed for static and dynamic canonical correlations, respectively. The relations between the EEG markers and AD severity were, in most cases, of non-monotonic nature with a slight increase for MMSE scores from 26 to 21, and a decrease below. During the cognitively active phase, the different synchrony markers corresponded better to the spacial cluster distribution: coherences and canonical correlations related significantly with MMSE scores between Central-Temporal/Left, partial coherences between Anterior-Central, the phase shift between Central-Posterior, and Granger causalities, conditional Granger causalities, and the cross-mutual information between several channel groups. The relations were stronger than in the resting phase, reaching $R^2$ values up to 0.462. Here too, ambiguous synchrony courses were observed with slopes that were generally steeper than during the resting phase. During both phases, our approach of synchrony calculation performed slightly better in terms of $R^2$ values than the averaging method by 0.06 and 0.08, respectively.

The analysis of the dependencies between the EEG synchrony markers revealed correlation patterns that were further investigated by common factor analysis. Factors were estimated by a maximum likelihood approach and oblique promax rotation. During the resting phase, coherences, partial coherences, ordinary and conditional (in direction $\rightarrow$) Granger causalities, and the phase shift in $\delta$ contributed positively to the first factor, whereas the phase shift in the remaining frequency bands contributed in a negative way. The second factor represented static and dynamic canonical correlations and, more weakly, the cross-mutual information. The first factor was most significantly related with MMSE scores between Anterior-Temporal/Left ($R^2 = 0.353$), Central-Temporal/Left ($R^2 = 0.332$), and Posterior-Temporal/Left ($R^2 = 0.271$). As for the individual synchrony markers, the first common factor scores followed an ambiguous trend with an increase for MMSE scores from 26 to 20 and a decrease below. During the active phase, coherences, ordinary and conditional (in direction $\rightarrow$) Granger causalities, static and dynamic canonical correlations, and the cross-mutual information contributed positively to the first factor, whereas the conditional Granger causality (in direction $\leftarrow$) contributed in a negative way. The second factor is mostly determined by the phase shift in a positive and by the partial coherences in a negative way. The first common factor showed highly significant relations with MMSE scores between Central-Temporal/Left ($R^2 = 0.433$) and Central-Temporal/Right ($R^2 = 0.302$). The reversed U-shape of the regression curve was much more distinct than during the resting phase.

### Comparison to state-of-the-art

The concepts of this work are, on first sight, similar to those of Dauwels et al. (2010b), Garn et al. (2015) and Garn et al. (2014). In Dauwels et al. (2010b), various EEG synchrony markers were used to distinguish patients suffering from mild cognitive impairment from age-matched control subjects. We used a different set of connectivity measures including conditional Granger causalities, static and dynamic canonical correlations. Whereas in (Dauwels et al. 2010b), the synchrony markers were used for assigning subjects to one of two groups, we addressed synchrony trends with cognitive decline as measured by neuropsychological test scores. In Dauwels et al. (2010b), 9 families of measures were observed by calculating the correlation coefficient between all pairs of spatially averaged synchrony measures. In this study, 3 families of resting phase measures [(1) (partial) coherences, Granger causalities; (2) phase shift, conditional Granger causalities; (3) (dynamic) canonical correlations, cross-mutual information] and 3 families of active phase measures [(1) coherences, Granger causalities, (dynamic) canonical correlations, cross-mutual information; (2) partial coherences, phase shift; (3) conditional Granger causalities) were identified. These findings differ from the measures of the families described in Dauwels et al. (2010b). However, in order to directly compare these findings, the same set of measures would have to be computed for the same electrodes. The cognitive task during EEG recording is another aspect that separates this study from Dauwels et al. (2010b), where the analysis of resting-state EEG was addressed. Finally, the estimation of common synchrony factors and relating them with AD severity separates the study approaches. Thus, on closer consideration, the perspectives of both studies differ in several aspects. In Garn et al. (2015), different EEG markers were used to describe major changes in the EEG of AD patients: relative spectral power in different frequency bands as markers for slowing, auto-mutual information and entropy as measures for reduced signal complexity, and, finally, coherences, Granger causalities, and canonical correlations as connectivity measures. In Garn et al. (2014), relative band powers, coherences, and auto-mutual information were applied to investigate whether memory paradigms during EEG recordings could improve the accuracy of diagnosing cognitive deficits. As compared to Garn et al. (2015) and Garn et al. (2014), this work provides a structured analysis of markers for EEG synchrony and the correlation patterns that they describe. A larger set of connectivity measures was applied including partial coherences, the phase shift, dynamic canonical correlations, and cross-mutual information. The focus on the comparison and combination of these measures introduced a novel perspective and new insights into the relation of EEG synchrony and AD severity. The analysis of oblique common synchrony factors that correspond to certain connectivity measures offered an alternative approach for studying connectivity in the EEG.

Other than that, there is only a small number of studies that are directly comparable to this work since most studies compare groups (e.g., healthy controls versus AD patients) instead of correlating EEG synchrony markers with AD severity. However, a major share of these group comparisons suggested a decrease of EEG synchrony in resting state (e.g., Dauwels et al. 2010a; Locatelli et al. 1998; Wada et al. 1998; Anghinah et al. 2000; Adler et al. 2003; Jelles et al. 2008; Babiloni et al. 2009; Wan et al. 2008; Stam et al. 2003; Pijnenburg et al. 2004; Kramer et al. 2007), and an increase during cognitive tasks for MCI and in few cases also for AD patients as compared to controls (e.g., Jiang 2005b; Jiang and Zheng 2006). This increase was attributed to compensatory mechanisms in the brain (cf. Dauwels et al. (2010b); Smith et al. 2007)). These synchrony changes were mostly reported for the left hemisphere, often between temporal and parietal, or temporal and central EEG sites. The mainly applied synchrony measures were coherences and, in more recent studies, non-linear measures originating from information theory. Studies that are directly comparable to this work reported no significant correlations between the degree of AD and coherences, neither in resting state (Adler et al. 2003; Kikuchi et al. 2002) nor during a working memory task (Kikuchi et al. (2002)). However, significant correlations were observed between the degree of AD and synchronization likelihood (Stam et al. 2003; Pijnenburg et al. 2004; Babiloni et al. 2006), and global field synchronization (Park et al. 2008), respectively.

We observed a synchrony increase in initial stages and a decrease in later stages of AD for most EEG markers and for the first common factors. This initial increase may be attributed to the same compensatory mechanisms in the brain that have been reported in Dauwels et al. (2010b), Park et al. (2008) and Smith et al. (2007). This phenomenon was most prominent during the cognitively active phase. In contrast to Adler et al. (2003) and Kikuchi et al. (2002), we observed significant changes of coherences during the active phase. This may be due to the applied quadratic regression that allowed to model ambiguous trends as well. The most significant changes of coherences were observed between Central-Temporal/Left; these findings correspond to the majority of group studies mentioned.

Apart from EEG synchrony markers, there is a large number of studies using markers for EEG slowing and reduced EEG complexity (c.f. Jeong 2004; Dauwels et al. 2010a; Garn et al. 2015). Especially the relative spectral power in the $\delta$-, $\theta$-, $\alpha$-, and $\beta$-frequency bands has been studied extensively in the context of AD. The aim of this work was to provide a detailed overview of EEG synchrony changes in AD; including additional slowing measures would have gone beyond the scope of this study. However, they have been reported for the same 79 AD subjects in Garn et al. (2015). A highly significant slowing of the EEG was observed especially in the $\theta$-band with $R^2$ values up to 0.51 in the left-hemispheric channels. More importantly, the changes in the $\delta$-, $\theta$-, and $\beta$-bands were of monotonic nature, whereas changes in the $\alpha$-band were non-monotonic as well, maybe due to same compensatory mechanisms as observed in this study.

### Strengths and limitations

The following paragraphs will discuss the strengths and limitations of this study. The EEG samples from the PRODEM-Austria database were all conducted in a uniform setting and according to a clinically predefined paradigm including both resting state and a cognitive task. The sample consisting of 79 EEG datasets from probable AD patients is, compared to the data in scientific literature, among the largest; a comparable or higher number has been reported in Wan et al. (2008) (103 AD, 124 controls), Babiloni et al. (2009) (73 AD, 69 subjects with MCI, 64 controls) and Babiloni et al. (2006) (109 AD, 88 subjects with MCI, 69 controls).

In this study, MMSE scores were used to quantify AD severity. Other comparable studies—e.g., Adler et al. (2003) and Stam et al. (2003)—have applied MMSE scores as well. However, MMSE and EEG do not necessarily measure the same cognitive processes. A variety of alternative neuropsychological assessments of cognitive impairment has been designed including the Clinical Dementia Rating Scale (CDR) Hughes et al. (1982), Disability Assessment for Dementia (DAD) Gélinas et al. (1999), Neuropsychiatric Inventory (NPI) Cummings et al. (1994), Geriatric Depression Scale (GDS) Yesavage et al. (1982–1983), and a neuropsychological test battery by the Consortium to Establish a Registry for AD (CERAD) Morris et al. (1989); Mirra et al. 1991). Investigating the relationship between the presented EEG synchrony markers and alternative neuropsychological instruments could provide additional insights into the neuronal and cognitive changes associated with AD severity.

We employed the demographic variables sex, age, level of education, and AD duration as co-variables. Age and level of education displayed a significant influence and explained approximately 22 % of the MMSE score variations. From the directly comparable studies listed above, Adler et al. (2003) and Pijnenburg et al. (2004) included the subjects’ age in the analysis. Park et al. (2008) accounted for age and level of education but detected no significant influences.

A crucial step in EEG analysis is the preprocessing procedure. Eliminating low-frequency artifacts by high-pass filtering is common practice in EEG analysis. The border frequency of 2 Hz was empirically determined. Algorithms for the detection and elimination of cardiac artifacts (cf. Waser and Garn (2013)) were applied and verified by visual examination. There is a broad range of alternative algorithms for the removal of cardiac artifacts, both relying solely on the EEG (e.g., Jiang et al. 2007; Jung et al. 2000) and relying on a simultaneously recorded ECG channel (e.g., Nakamura and Shibasaki 1987; Park et al. 1998). For the removal of eye artifacts, the EOG channels were utilized. Other procedures (often in the absence of EOG channels) such as blind source separation have been applied in several studies, e.g., in Jung et al. 2000. In the final preprocessing step, the EEG data were low-pass filtered. The border frequency of 15 Hz was determined by comparing the spectra of channels with and without muscular artifacts. The EEG recordings were equally divided into segments of 4 seconds with an overlap of 2 seconds. Adaptive segmentation procedures have been described as alternative approach in e.g., Bodenstein and Praetorius (1977) and Deistler et al. (1986). However, these procedures require structural breaks in the data, e.g., when the patient opens their eyes. Within the EEG phases, no severe structural breaks were observed and, thus, the uniform length segments applied. The stationarity of the 4-second segments was verified by an augmented Dickey–Fuller test Dickey and Fuller (1979).

Dividing the frequency domain in frequency bands is common practice in EEG analysis; however, frequency borders vary in literature and the transition frequencies between the four frequency bands may differ from the transition frequencies used here by $\pm$1 Hz. The lower frequency border of the $\delta$-band is often defined as 0 or 0.5 Hz. The upper $\beta$-border is usually defined in a range of 20 to 30 Hz. We are aware that the low border of 15 Hz introduces neurophysiological limitations since the frequency range above 15 Hz is associated with a variety of cognitive functions including concentration and stimuli of the motor cortex. However, these limitations were accepted in order to make sure that no artifacts deteriorate the analyses. An alternative to fixed frequency bands would be an individualization by means of the position of spectral peaks such as the individual alpha frequency, as well as the transition frequencies between these peaks. As these peaks and the transition between them vary widely amongst different subjects, electrode channels, and cognitive phases, an individualization of frequency bands is a non-trivial task that would have complicated the analyses.

All but one synchrony marker were derived from the spectral density and are, in a certain sense, of linear nature. Due to the complexity of neuronal processes, recent studies have considered non-linear measures including mutual information Jeong et al. (2001), synchronization likelihood Stam et al. 2003; Pijnenburg et al. 2004; Babiloni et al. 2006, global field synchronization Park et al. (2008), and global synchronization index Li et al. 2007; Lee et al. 2010. We observed highly significant relations between cross-mutual information and AD severity as well. These results suggest that non-linear markers may be able to contribute valuable information to any synchrony analysis. Combining linear and non-linear synchrony markers could considerably improve the understanding of EEG synchrony changes in AD.

With the aim of gaining robustness, we estimated the synchrony markers between clusters of EEG channels instead of between single electrode sites. This approach seems reasonable since changes in the EEG reflect functional changes in the cortical areas beneath the electrodes Jeong (2004). By arranging the electrodes in clusters corresponding to the cerebral lobe structure, we tried to describe the patterns of these functional changes more accurately. For each cluster, we conducted PCA and investigated the maximum synchrony between the PCs of two clusters under consideration. Only the first two PCs of one cluster and the first two PCs of another cluster were hereby used, since they already accounted for over 90 % of the variability in the respective channel data. This insight suggested a two-dimensional static structure which was indicative for a high degree of homogeneity within a cluster. This method has already been demonstrated in Garn et al. (2015), Garn et al. (2014) and (Waser et al. , 2013). However, PCs accounting for low portions of variability may still have a substantial functional significance and should be further investigated. The authors did not intend to attribute physical meaning to the individual PCs. Alternative approaches besides PCA include the computation of EEG markers between EEG channel pairs and averaging over all these pairs (c.f. Dauwels et al. 2010b). In this study, our PCA approach performed on average better in terms of $R^2$ values than the averaging method, thus indicating slightly more robustness. This may be due to the 19-channel framework with its rough spatial resolution. High-density EEG recordings with a higher number of channels would allow for even more homogeneous clusters that correspond better to the cerebral lobe structure.

For diagnostic purposes, a steep monotonic synchrony trend with decreasing MMSE would be preferable. Our synchrony markers, however, displayed non-monotonic courses with decreasing MMSE scores. The employment of quadratic regression models for the description of synchrony changes allowed us to capture these non-monotonic trends that could be reflecting compensatory brain mechanisms (Park et al. 2008; Dauwels et al. 2010b; Smith et al. 2007). The reversed U-shaped trends bring diagnostic ambiguity with them. None of the individual EEG markers were capable of distinguishing patients above and below MMSE scores of 21, and a classification based on a combination of all markers did not yield satisfying results in terms of sensitivity and specificity. However, especially during the cognitive task, rather steep slopes both for high and low MMSE scores were observed. Thus, these synchrony markers could provide information for characterizing AD severity in subgroups of patients where the approximate stage of cognitive decline is known a priori. However, all these relations were obtained for the overall patient group; they were not strong enough to be suited as stand-alone criterion for individual diagnosis. A combination of the presented synchrony markers with markers for slowing or reduced EEG complexity with potentially more monotonic changes could help in refining this criterion.

### Conclusion

In conclusion, this study indicates that several of the presented synchrony markers relate to AD severity as measured by MMSE scores. The accumulation of channels allowed a robust analysis of synchrony. The most prominent significant results were observed between anterior-temporal and posterior-temporal electrode sites during the resting phase, and between anterior-central, central-posterior, and central-temporal sites during the cognitively active phase. The different markers—although closely related to each other—captured different aspects of EEG synchrony. Estimating common factors and relating these factors with AD severity was demonstrated to be an alternative approach to using individual markers only. Using demographic co-variables led to an improvement of the analysis. Another key aspect was the use of quadratic regression instead of commonly used linear regression models. This approach allowed to capture ambiguous trends as well. Most markers displayed an initial increase of EEG synchrony (MMSE $>$20) and a decrease in later stages. This effect was most prominent in the cognitive phase and may be owed to compensatory brain mechanisms. Although this phenomenon causes diagnostic ambiguity, its analysis may provide supplementary information for understanding the neuronal changes in AD. Especially during the cognitively active phase, the slope of the estimated synchrony course was steep both at high and low MMSE scores and could help in the diagnostics of patients where the approximate stage of cognitive decline is already known. However, we should also remark that all these relations were obtained for the overall patient group and that they were not strong enough to be suited as stand-alone criterion for individual diagnosis. Part of the variations in the scatter diagrams may be caused by fluctuations associated with MMSE measurements.

Future studies should both relate the presented EEG synchrony markers with alternative neuropsychological assessments of AD severity and combine them with other EEG markers that capture changes in signal complexity and frequency content as well. Longitudinal studies need to determine as to whether the EEG markers can help in describing AD progression. The combination of EEG markers with other potential structural and functional AD markers could then aid in the diagnostics of AD.

## Appendix: Result tables

See Appendix Table 2 and 3.

**Table 2:** Coefficients of determination $R^2$ for all EEG synchrony markers and all cluster pairs in the resting phase. Significant results after Bonferroni correction are represented in a bold font

EEG marker		A-C	A-P	A-TL	A-TR	C-P	C-TL	C-TR	P-TL	P-TR	TL-TR
*C*											
$\delta$		0.121	0.139	**0.314**	0.201	0.203	**0.264**	**0.271**	**0.277**	**0.344**	0.108
$\theta$		0.143	0.119	0.248	0.219	0.165	0.242	0.233	**0.286**	**0.321**	0.158
$\alpha$		0.128	0.168	0.210	0.191	0.139	0.251	0.238	0.237	**0.257**	0.126
$\beta _0$		0.100	0.251	0.239	0.190	0.160	0.214	0.166	0.189	0.209	0.147
*pC*											
$\delta$		0.214	**0.290**	0.191	**0.260**	0.149	0.136	0.169	0.223	0.202	**0.302**
$\theta$		0.187	**0.311**	**0.284**	**0.280**	0.188	0.193	0.190	**0.291**	0.252	**0.258**
$\alpha$		0.172	0.204	0.175	0.127	0.188	0.163	0.226	**0.318**	0.250	**0.286**
$\beta _0$		0.228	0.147	**0.326**	0.176	0.199	0.093	0.158	**0.256**	0.211	0.176
$$n\Phi $$											
$\delta$		0.153	0.205	0.246	0.241	0.217	0.179	0.148	0.127	0.176	0.188
$\theta$		0.149	0.222	0.242	0.239	0.248	0.183	0.165	0.162	0.196	0.214
$\alpha$		0.143	0.250	**0.350**	**0.295**	0.242	0.212	0.194	0.147	0.199	0.165
$\beta _0$		0.131	0.165	**0.257**	**0.261**	0.158	0.183	0.156	0.133	0.210	0.171
*G*											
$\rightarrow$		0.176	0.235	**0.374**	**0.275**	0.208	**0.291**	0.188	0.181	0.247	0.195
$\leftarrow$		0.191	0.174	**0.272**	0.144	0.221	0.158	0.214	0.254	**0.260**	0.135
*cG*											
$\rightarrow$		**0.327**	**0.275**	**0.281**	0.230	0.193	0.194	0.166	**0.269**	**0.313**	0.147
$\leftarrow$		0.199	**0.301**	0.189	0.221	**0.256**	0.217	0.239	**0.257**	**0.272**	0.152
$\rho ^c$		0.113	0.065	0.104	0.087	0.071	0.108	0.135	0.092	0.066	0.088
$$d\rho ^c$$											
$\delta$ $\theta$		0.125	0.109	0.162	0.169	0.120	0.222	**0.316**	0.155	0.198	0.168
		0.102	0.162	0.080	0.149	0.109	0.064	0.140	0.154	0.207	0.120
$\alpha$		0.132	0.080	0.023	0.097	0.119	0.042	0.211	0.076	0.053	0.084
$\beta _0$		0.146	0.140	0.149	0.127	0.212	0.035	0.148	0.148	0.080	0.107
*cMI*		**0.303**	0.237	**0.351**	0.231	**0.360**	0.249	**0.257**	**0.306**	**0.386**	**0.345**

**Table 3:** Coefficients of determination $R^2$ for all EEG synchrony markers and all cluster pairs in the active phase. Significant results after Bonferroni correction are represented in a bold font

EEG marker		A-C	A-P	A-TL	A-TR	C-P	C-TL	C-TR	P-TL	P-TR	TL-TR
*C*											
$\delta$		0.213	0.156	0.233	0.164	0.142	**0.399**	0.195	0.163	**0.260**	**0.283**
$\theta$		0.214	0.080	0.234	0.172	0.133	**0.366**	0.140	0.135	0.213	0.250
$\alpha$		**0.270**	0.182	0.194	0.226	0.125	**0.393**	**0.279**	0.183	0.130	0.171
$\beta _0$		0.214	**0.285**	0.163	0.257	0.154	0.223	0.176	0.203	0.135	0.214
*pC*											
$\delta$		**0.344**	0.104	0.210	0.181	0.201	0.165	0.186	0.143	0.193	0.143
$\theta$		**0.420**	0.184	0.116	0.214	**0.272**	0.178	0.141	0.170	0.161	0.225
$\alpha$		**0.462**	0.109	0.099	0.157	0.174	0.166	0.185	0.105	0.167	0.157
$\beta _0$		**0.350**	0.246	0.101	0.081	0.188	0.129	0.208	0.087	0.202	0.166
$$n\Phi $$											
$\delta$ $\theta$		0.255	0.215	**0.310**	0.230	**0.261**	0.241	0.098	0.208	0.238	0.219
		0.196	0.230	**0.283**	0.245	**0.274**	**0.284**	0.121	0.175	0.237	0.233
$\alpha$		0.176	0.237	0.243	0.208	**0.261**	0.151	0.136	0.150	0.253	0.199
$\beta _0$		0.169	**0.289**	0.165	0.198	**0.272**	0.143	0.138	0.115	0.231	0.186
*G*											
$\rightarrow$		**0.354**	0.230	**0.273**	0.193	**0.329**	**0.307**	**0.289**	**0.377**	0.184	0.234
$\leftarrow$		0.199	0.142	0.147	0.160	**0.277**	0.169	0.169	0.223	0.150	0.222
*cG*											
$\rightarrow$		**0.274**	0.181	0.231	0.176	0.223	0.156	**0.266**	**0.321**	0.200	**0.304**
$\leftarrow$		0.142	0.139	0.233	0.130	**0.316**	0.174	0.197	0.241	0.244	0.193
$\rho ^c$		0.164	0.139	0.238	0.141	0.104	**0.402**	0.225	**0.263**	0.167	0.200
$$d\rho ^c$$											
$\delta$		0.138	0.112	0.089	0.105	0.121	0.185	0.258	0.148	0.051	0.174
$\theta$		0.225	**0.264**	0.253	0.125	0.150	**0.366**	0.220	0.226	0.222	0.152
$\alpha$		0.173	0.052	0.048	0.124	0.101	0.218	0.168	0.172	0.165	0.098
$\beta _0$		0.094	0.107	0.133	0.160	0.121	0.085	0.175	0.246	0.135	0.063
*cMI*		**0.288**	0.255	0.216	**0.269**	**0.386**	**0.373**	**0.329**	**0.294**	**0.363**	**0.368**

## Footnotes

1 Here, the control group consisted of patients suffering from depression.

2 The applied FIR filter design method was based on ideal filter approximation using a window function, where the approximation error decreased with increasing filter order. We used zero-phase forward and reverse digital filtering, where the filter order is limited to a third of the sampling points by the length of edge transients. Since the minimum length of EEG segments was 1024 sampling points (i.e., 4 s), the maximum filter order was $\frac{1024}{3}\approx 341$. The software implementation required an even filter order, thus leading to an order of 340.

## References

[CR1] Adler G, Brassen S, Jajcevic A (2003). EEG coherence in Alzheimer’s dementia. J Neural Trans.

[CR2] Akaike H (1974). A new look at the statistical model identification. IEEE Trans Automatic Control.

[CR3] Akrofi K et al. (2009) A Model of Alzheimer’s disease and mild cognitive impairment based on EEG coherence. ICME International Conference on Complex Medical Engineering pp 1–6

[CR4] Alzheimer’s Disease International (2010) World Alzheimer Report 2010: The global economic impact of dementia

[CR5] Alzheimer’s Disease International (2011) World Alzheimer Report 2011: the benefits of early diagnosis and intervention

[CR6] Alzheimer’s Disease International (2013) Policy brief for heads of government: the global impact of dementia 2013–2050

[CR7] Anghinah R (2000). Alpha band coherence analysis of EEG in healthy adult and Alzheimer’s type dementia subjects. Arquivos de Neuro-Psiquiatria.

[CR8] Babiloni C (2006). Fronto-parietal coupling of brain rhythms in mild cognitive impairment: a multicentric EEG study. Brain Res Bull.

[CR9] Babiloni C (2009). Directionality of EEG synchronization in Alzheimer’s disease subjects. Neurobiol Aging.

[CR10] Bodenstein G, Praetorius HM (1977). Feature extraction from the electroencephalogram by adaptive segmentation. Proceed IEEE.

[CR11] Braak H et al (2006) Vulnerability of cortical neurons to Alzheimer’s and Parkinson’s diseases. Alzheimer’s disease: a century of scientific and clinical research pp 35–44 10.3233/jad-2006-9s305 16914843

[CR12] Bracco L (1994). Factors affecting course and survival in Alzheimer’s disease: a 9-year longitudinal study. Archiv Neurol.

[CR13] Brillinger DR (1981) Time series: data analysis and theory. Holden-Day

[CR14] Carroll JB (1953). An analytical solution for approximating simple structure in factor analysis. Psychometrika.

[CR15] Cummings JL, Mega M, Gray K, Rosenberg-Thompson S, Carusi DA, Gornbein J (1994). The Neuropsychiatric inventory: comprehensive assessment of psychopathology in dementia. Neurology.

[CR16] Dahlhaus R (2000). Graphical interaction models for multivariate time series. Metrika.

[CR17] Dauwels J, Vialatte F, Cichocki A (2010). Diagnosis of Alzheimer’s disease from EEG signals: where are we standing?. Curr Alzheimer Res.

[CR18] Dauwels J et al (2007) Measuring neural synchrony by message passing. Advances in neural information processing systems, p 20

[CR19] Dauwels J (2010). A comparative study of synchrony measures for the early diagnosis of Alzheimer’s disease based on EEG. NeuroImage.

[CR20] Deistler M (1986). Procedure for identification of different stages of EEG background activity and its application to the detection of drug effects. Electroencephal Clinical Neurophysiol.

[CR21] Dickey DA, Fuller WA (1979). Distribution of the estimators for autoregressive time series with a unit root. J Am Statist Assoc.

[CR22] Eichler M (2006) Graphical modeling of dynamic relationships in multivariate time series. In: Handbook of time series analysis. Wiley-VCH, pp 335–372

[CR23] Flamm C et al (2012) Graphs for dependence and causality in multivariate time series. System identification, environmental modelling, and control system design. Springer, London, pp 133–151

[CR24] Folstein MF, Folstein SE, McHugh PR (1975). ’Mini-mental state’. A practical method for grading the cognitive state of patients for the clinician. J Psychiat Res.

[CR25] Garn H et al (2014) Quantitative EEG in Alzheimer’s disease: cognitive state, resting state and association with disease severity. International Journal of Psychophysiology pp 390–397 10.1016/j.ijpsycho.2014.06.003 24933410

[CR26] Garn H (2015). Quantitative EEG markers relate to Alzheimer’s disease severity in the Prospective Dementia Registry Austria (PRODEM). Clin Neurophysiol.

[CR27] Gélinas I, Gauthier L, McIntyre M (1999). Development of a functional measure for persons with Alzheimer’s disease: the Disability Assessment for Dementia. Am J Occup Therapy.

[CR28] Granger CWJ (1969). Investigating causal relations by econometric models and cross-spectral methods. Econometrica.

[CR29] Güntekin B, Saatçi E, Yener G (2009). Decrease of evoked delta, theta and alpha coherences in Alzheimer patients during a visual oddball paradigm. Brain Res.

[CR30] Hannan EJ, Deistler M (1988) The statistical theory of linear systems. Wiley

[CR31] Hidasi Z (2007). Changes of EEG spectra and coherence following performance in a cognitive task in Alzheimer’s disease. International journal of psychophysiology.

[CR32] van der Hiele K (2007). EEG correlates in the spectrum of cognitive decline. Clinical neurophysiology.

[CR33] Hogan MJ (2003). Memory-related EEG power and coherence reductions in mild Alzheimer’s disease. International journal of psychophysiology.

[CR34] Hotelling H (1936). Relations Between Two Sets of Variates. Biometrika.

[CR35] Hsu JC (1996) Multiple comparisons—theory and methods. Chapman & Hall London

[CR36] Hughes CP, Berg L, Danziger WL, Coben LA, Martin RL (1982). A new clinical scale for the staging of dementia. The British journal of psychiatry.

[CR37] Jasper HH (1958). The ten-twenty electrode system of the International Federation. Electroencephalography and clinical neurophysiology.

[CR38] Jelles B (2008). Global dynamical analysis of the EEG in Alzheimer’s disease: frequency-specific changes of functional interactions. Clinical neurophysiology.

[CR39] Jellinger KA (2007). The enigma of mixed dementia. Alzheimer’s and dementia.

[CR40] Jeong J (2004). EEG dynamics in patients with Alzheimer’s disease. Clinical neurophysiology.

[CR41] Jeong J, Gore J, Peterson B (2001). Mutual information analysis of the EEG in patients with Alzheimer’s disease. Clinical neurophysiology.

[CR42] Jiang JA (2007). An automatic analysis method for detecting and eliminating ECG artifacts in EEG. Computers in Biology and Medicine.

[CR43] Jiang ZY (2005). Abnormal Cortical Functional Connections in Alzheimer’s Disease: Analysis of Inter- and Intra-Hemispheric EEG Coherence. Journal of Zhejiang University science B.

[CR44] Jiang ZY (2005). Study on EEG Power and Coherence in Patients with Mild Cognitive Impairment During Working Memory Task. Journal of Zhejiang University science B.

[CR45] Jiang ZY, Zheng LL (2006). Inter- and intra-hemispheric EEG coherence in patients with mild cognitive impairment at rest and during working memory task. Journal of Zhejiang University science B.

[CR46] Jung TP (2000). Removing electroencephalographic artifacts by blind source separation. Psychophysiology.

[CR47] Kaplan AY (2005). Nonstationary nature of the brain activity as revealed by EEG/MEG: Methodological, practical and conceptual challenges. Signal processing.

[CR48] Kikuchi M (2002). EEG harmonic responses to photic stimulation in normal aging and Alzheimer’s disease: differences in interhemispheric coherence. Clinical neurophysiology.

[CR49] Kramer M (2007). Synchronization measures of the scalp EEG can discriminate healthy from Alzheimer’s subjects. International journal of neural systems.

[CR50] Laske C (2015). Innovative diagnostic tools for early detection of Alzheimer’s disease. Alzheimer’s and dementia.

[CR51] Lawley DN (1940). The estimation of factor loadings by the method of maximum likelihood. Proceedings of the Royal Society of Edinburgh.

[CR52] Lee SH, Park YM, Kim DW, Im CH (2010). Global synchronization index as a biological correlate of cognitive decline in Alzheimer’s disease. Neuroscience Research.

[CR53] Li X (2007). Synchronization measurement of multiple neuronal populations. Journal of Neurophysiology.

[CR54] Locatelli T (1998). EEG coherence in Alzheimer’s disease. Electroencephalography and clinical neurophysiology.

[CR55] Maes F (1997). Multimodality image registration by maximization of mutual information. IEEE Transactions on Medical Imaging.

[CR56] Mann HB, Whitney DR (1947). On a test of whether one of two random variables is stochastically larger than the other. Annals of Mathematical Statistics.

[CR57] McKhann G (2011). The diagnosis of dementia due to Alzheimer’s disease: Recommendations from the National Institute on Aging-Alzheimer’s Association workgroups on diagnostic guidelines for Alzheimer’s disease. Alzheimer’s and dementia.

[CR58] Mirra S, Heyman A, McKeel D, Sumi SM, Crain BJ, Brownlee LM (1991). The Consortium to Establish a Registry for Alzheimer’s Disease (CERAD): part 2 clinical and neuropsychological assessment of Alzheimer’s disease. Neurology.

[CR59] Morris JC, Heyman A, Mohs RC, Hughes JP, van Belle G, Fillenbaum G (1989). The Consortium to Establish a Registry for Alzheimer’s Disease (CERAD): part 1 clinical and neuropsychological assessment of Alzheimer’s disease. Neurology.

[CR60] Nakamura M, Shibasaki H (1987). Elimination of EKG artifacts from EEG recordings: a new method of noncephalic referential EEG recording. Electroencephalography and Clinical Neurophysiology.

[CR61] Park HJ, et al (1998) A study on the elimination of the ECG artifact in the polysomnographic EEG and EOG using AR model. In: 20th Annual International Conference of the IEEE EMBS, pp 1632–1635

[CR62] Park YM (2008). Decreased EEG synchronization and its correlation with symptom severity in Alzheimer’s disease. Neuroscience research.

[CR63] Parzen E (1962). On Estimation of a Probability Density Function and Mode. The annals of mathematical statistics.

[CR64] Pijnenburg YA (2004). EEG synchronization likelihood in mild cognitive impairment and Alzheimer’s disease during a working memory task. Clinical neurophysiology.

[CR65] Pijnenburg YA (2008). Investigation of resting-state EEG functional connectivity in frontotemporal lobar degeneration. Clinical neurophysiology.

[CR66] Schmidt R (2010). Consensus statement ’Dementia 2010’ of the Austrian Alzheimer Society. Neuropsychiatry.

[CR67] Shannon CE, Weaver W (1949). The mathematical theory of communication.

[CR68] Smith GE (2007). A plateau in pre-Alzheimer memory decline: Evidence for compensatory mechanisms?. Neurology.

[CR69] Stam CJ, Nolte G, Daffertshofer A (2007). Phase lag index: assessment of functional connectivity from multi channel EEG and MEG with diminished bias from common sources. Human brain mapping.

[CR70] Stam CJ (2003). EEG synchronization in mild cognitive impairment and Alzheimer’s disease. Acta neurologica scandinavica.

[CR71] Stam CJ (2005). Disturbed fluctuations of resting state EEG synchronization in Alzheimer’s disease. Clinical neurophysiology.

[CR72] Stevens A (2001). Dynamic regulation of EEG power and coherence is lost early and globally in probable DAT. European archives of psychiatry and clinical neuroscience.

[CR73] Tukey JW (1967) An introduction to the calculations of numerical spectrum analysis. Spectral Anal Time Series pp 25–46

[CR74] Wada Y (1998). Reduced interhemispheric EEG coherence in Alzheimer’s disease: analysis during rest and photic stimulation. Alzheimer disease and associated disorders.

[CR75] Walker G (1931). On periodicity in series of related terms. Proceedings of the Royal Society of London, Ser A.

[CR76] Wan B (2008). Linear and nonlinear quantitative EEG analysis. IEEE engineering in medicine and biology magazine.

[CR77] Waser M, Garn H (2013) Removing cardiac interference from the electroencephalogram using a modified Pan-Tompkins algorithm and linear regression. In: 35th Annual International Conference of the IEEE EMBS, pp 2028–2031 10.1109/EMBC.2013.6609929 24110116

[CR78] Waser M (2013). EEG in the diagnostics of Alzheimer’s disease. Statistical Papers.

[CR79] Waser M, et al (2014) Using Static and Dynamic Canonical Correlation Coefficients as Quantitative EEG Markers for Alzheimer’s Disease Severity. In: 36th Annual International Conference of the IEEE EMBS, pp 2801–2804 10.1109/EMBC.2014.6944205 25570573

[CR80] World Health Organization and Alzheimer’s Disease International (2012) Dementia: A public health priority

[CR81] Yesavage JA, Brink TL, Rose TL, Lum O, Huang V, Adey M, Leirer VO (1982). –1983) Development and validation of a geriatric depression screening scale: a preliminary report. Journal of psychiatric research.

[CR82] Yule GU (1927). On a method of investigating periodicities in disturbed series, with special reference to Wolfer’s sunspot numbers. Philosophical Transactions of the Royal Society of London, Ser A.

